# Use of extended reality, virtual reality, augmented reality and mixed reality in clinical practice, education and research in knee arthroplasty: A scoping review

**DOI:** 10.1002/jeo2.70788

**Published:** 2026-06-16

**Authors:** Ritesh Zun Xiong Deo, Trishan Manav Sri Ram, Hamid Rahmatullah Bin Abd Razak, Nanne Kort

**Affiliations:** ^1^ Woodlands Health Campus, National Healthcare Group Singapore Singapore; ^2^ Yong Loo Lin School of Medicine National University of Singapore Singapore Singapore; ^3^ Total Orthopaedic Care and Surgery Singapore Singapore; ^4^ Cortoclinics Nederweert the Netherlands

**Keywords:** augmented reality, extended reality, knee arthroplasty, mixed reality, virtual reality

## Abstract

**Purpose:**

This scoping review aims to map and evaluate the current body of literature on the use of extended reality (XR), including virtual reality (VR), augmented reality (AR) and mixed reality (MR), in the field of knee arthroplasty. There is a high global prevalence of knee osteoarthritis, and the frequency of knee replacement surgeries is increasing. The integration of XR technologies with surgery has the potential to improve patient care, surgical precision and medical education. This review seeks to understand the current landscape of XR applications in knee arthroplasty and identify gaps in knowledge to guide future research and clinical innovation.

**Methods:**

A systematic search of four databases—PubMed, Cochrane Central Register for Controlled Trials, National University of Singapore Libraries and Google Scholar—was conducted in December 2023 and updated in April 2024. Only English‐language articles published from 2004 onwards were included. Editorials, case reports and articles not related to the knee joint were excluded. Eligible studies involved the use of XR/VR/AR/MR technologies specifically in the context of knee arthroplasty. Included articles were categorised under three major themes: (1) Clinical Practice (encompassing surgery, anaesthesia and rehabilitation), (2) Education (targeting both surgeons and nursing staff) and (3) Research (including applications in artificial intelligence and robotic‐assisted surgery). Data from each study were extracted and summarised in a thematic table.

**Results:**

Out of 236 articles retrieved from databases and 5 identified through reference screening, 54 articles met the inclusion criteria. VR was the most commonly studied modality (*n *= 42), followed by AR (*n *= 21), MR (*n *= 5), and XR (*n *= 2), with some overlap (*n *= 9) across technologies. Most articles focused on clinical practice (*n *= 45), while fewer addressed educational uses (*n *= 9) and research applications (*n *= 2). Two studies were classified under multiple themes. XR technologies were applied across preoperative training, intraoperative surgical navigation, anaesthesia techniques and postoperative rehabilitation.

**Conclusions:**

XR technologies in knee arthroplasty are diverse and show promising applications across clinical, education and research domains. While surgery, anaesthesia and education‐related applications appear practical and beneficial, rehabilitation‐related studies report mixed outcomes. Further high‐quality research is needed to evaluate effectiveness and support broader clinical implementation across all identified subthemes.

**Level of Evidence:**

Level V.

**Abbreviations**
AI: artificial intelligence
AR: augmented reality
COVID‐19: coronovirus disease 2019
CT: computed tomography
CV: computer vision
HMD: head‐mounted displays
iVR: immersive virtual reality
MESH: medical subject headings
NUS: National University of Singapore
OA: osteoarthritis
OSATS: objective structured assessment of technical skills
TKA: total knee arthroplasty
VR: virtual reality
XR: extended reality

## INTRODUCTION

**Figure 1:**
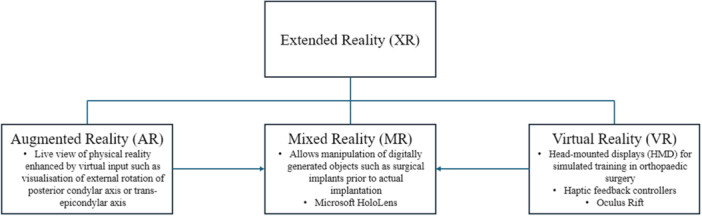
XR is an umbrella term that encompasses AR, VR and MR. All XR involves an immersive experience for the user that could solely be a completely different reality (VR), a supplemented reality (AR) or a combination of the interactive function with a supplemented reality. (MR) [119] Each technology is supplemented with a real‐world application example. AR, augmented reality; MR, mixed reality; VR, virtual reality; XR, extended reality.

In healthcare, the culmination of advanced computing and imaging technologies has led to the creation of a spectrum of extended reality (XR) technology—encompassing VR, augmented reality (AR) and mixed reality (MR) (Figure 1) [69, 119]. The use of XR in surgical subspecialties was initially described in 1998 [18] and has been increasingly pervasive due to its multifunctionality preoperatively, intraoperatively and postoperatively [125, 132]. XR technology has multiple use cases in the field of healthcare and can be categorised broadly into categories of education (for doctors and medical students), cardiac applications (such as intraprocedural visualisation), surgery (such as presurgical planning), neurological disorders (such as stroke rehabilitation or teaching social skills to children with attention deficit hyperactivity disorder), dental medicine (such as maxilla‐facial surgery), orthopaedics and biomedical trends (such as biomedical devices and telemedicine) [120].

Osteoarthritis (OA) is a progressive and degenerative disease that involves all joints due to degenerative changes in cartilage secondary to biomechanical joint stress and biochemical changes [46]. In the global ageing population, it is the most common disease of the musculoskeletal system [138] and affects 70% of women and 60% of men above 65 years old [116]. The knee is affected in 82.6% of OA cases [31]. Risk factors for OA include age, obesity, and knee overuse through physical activity [135]. Knee OA patients may experience a poorer quality of life with limited daily activities. Signs and symptoms of knee OA include articular pain, stiffness, crepitus, bony enlargement and reduced mobility with functional and balance disorders [13, 75, 127].

The conservative management for knee OA includes physiotherapy, function improvement and analgesia [45]. When conservative management for knee OA has proven futile, total knee arthroplasty (TKA) is a standard therapeutic and surgical option [7, 17, 48, 67, 107, 117, 137]. There has been an increasing number of knee OA patients and patients undergoing knee arthroplasty over the years [36, 59, 101, 125]. The goal of TKA is to improve the pain, function, strength and range of motion of the knee. This could lead to improved quality of life for OA knee patients [45, 52, 125]. The success of knee arthroplasty is influenced by various factors such as post‐operative rehabilitation and exercise, surgical skills, patient expectations, implant design and individual target definition [3, 40]. The application of XR technology in knee arthroplasty aims to improve these factors and ultimately improve patient outcomes.

VR may be categorised as immersive, nonimmersive and semi‐immersive. These categories can be distinguished based on the number of senses engaged, the degree of interaction with the virtual milieu, virtual stimuli reliability and the user's disparity from external stimuli (e.g., room light) [12, 124]. VR may also be classified based on its interactivity—noninteractive, interactive with visual feedback or interactive with haptic feedback [90].

Non‐immersive VR involves the projection of a two‐dimensional overlay on a screen with the possibility of interaction with the overlay via a controller such as a keyboard or a joystick. Nonimmersive VR is generally cheaper and more readily available than immersive VR [95, 96].

Immersive VR (iVR) optically immerses the user in a synthetic and digital three‐dimensional environment by blocking out external stimuli [10, 53, 69, 74, 125, 126, 132]. iVR might include the use of head‐mounted displays (HMD), such as the Oculus Rift (Meta) and haptic feedback via controllers to enhance the holistic, immersive experience [50, 109, 119]. Despite having its roots in the aviation industry and the military [66], the use of iVR in surgery dates to as early as 1988 [22] and has recently gained popularity [50, 118]. iVR allows users to rehearse and practice procedures before entering the operating theatre [29] and acts as an educational resource to learn new surgical techniques without incurring risks of harm to patients, without the use of expensive resources (e.g., cadavers) or the need for supervision [62, 78, 88]. Furthermore, iVR has served as a form of distraction for patients to decrease anxiety and discomfort during procedures such as wound care, physiotherapy, dental and perioperative procedures such as nerve blocks or anaesthesia induction [43, 44, 61, 91, 100, 106] or even as an adjunct intraoperatively for regional/neuraxial anaesthesia [63, 73, 134].

AR employs smart devices with wearable headsets to superimpose an artificial three‐dimensional overlay (such as video, graphics or global positioning system data) onto real‐world surfaces to assimilate virtual objects with real environments [6, 37, 69, 74, 93, 125, 126, 132, 143]. There has been an increasing interest in the utility of AR in surgery [140]. The benefits of AR include enhancing the surgeon's focus on the surgical field, decreasing surgical field barriers and reducing dangerous radiation exposure [16, 99]. AR‐based navigation technologies have been proven to improve component positioning in TKA [130]. Furthermore, AR‐based rehabilitation has improved intraoperative proprioception by promoting real‐time interaction between real and virtual environments [122].

MR technology involves the utilisation of both AR and VR combined, encompassing aspects of both real and VR, such as the MR‐based Microsoft HoloLens [69, 74, 119, 125, 126, 132]. MR exists along a virtual continuum as a spectrum with the digital world on one end and the physical world on the other [119]. It relies on AI systems, software processing, display output and cloud computing, facilitating control of virtual objects (via VR) while simultaneously having an artificial overlay onto reality (via AR) [119]. MR allows for more customisability in preoperative planning than CT reconstructions alone. It provides intraoperative visualisation and data manipulation, such as manipulating virtual overlays of surgical implants over the surgical field before implantation [119, 132].

XR technology aims to provide a cost‐effective and time‐saving method of training. It allows for simulation training sessions before real‐world surgical practice, reducing operative complications and risks [69]. XR technology also empowers patients to be more involved in the care of their disease. This is achieved through improvements in patient health awareness, increased compliance with treatment and increased patient satisfaction [85]. XR is proposed to reduce the cost of knee arthroplasty. This is achieved by removing the need for imaging (such as preoperative fluoroscopy) and the use of expensive equipment or machinery (such as robot‐assisted or computer navigation surgery). These requirements are superseded by XR's ability to utilise data visualisation in diagnostic and therapeutic procedures [119].

The objective of this scoping review is to map out the current literature on the emerging topic of using XR, VR, AR and MR in the different aspects of knee arthroplasty. This review will explore how each technology is used and identify gaps to guide future research in this field.

## METHODS

This scoping review aims to summarise the existing literature concerning the use of XR, VR, AR and MR in clinical practice, education and research in knee arthroplasty. This scoping review was performed in a systematic approach broadly in keeping with the framework for conducting scoping reviews by Arksey and O'Malley (enhanced by Daudt, Van Mossel and Scott [35]) and the extended preferred reporting items for systematic reviews and meta‐analysis for scoping reviews (PRISMA‐ScR) [2, 128]. The primary research question was to map out the current use of XR, VR, AR and MR in clinical practice, education and research in knee arthroplasty. This was developed in keeping with the population, concept and context model for conducting scoping review questions [103].

The literature review was focused on articles written in English that included the use of XR and/or VR, and/or AR, and/or MR in the use of knee arthroplasty. Articles had to include a general definition or explanation of the technology used (XR, VR, AR or MR).

Articles were excluded if they were published before 2004. This timeframe was chosen because it reflects the global advent of XR, VR, AR and MR applications. The exclusion criteria were: (1) editorial articles, (2) case reports or (3) studies evaluating joints apart from the knee. Articles were also excluded if there was no access to the complete publication.

A comprehensive literature review included keywords and Medical Subject Headings (MESH terms) for title and abstract screening. The databases used were PubMed, Cochrane Central Register for Controlled Trials, NUS Libraries and Google Scholar. The search was conducted in December 2023 and updated in April 2024. The articles were compared against the inclusion and exclusion criteria for significance.

Following the review, articles were categorised into themes of clinical practice, education and research in knee arthroplasty. Relevant articles were stored and organised using Mendeley (reference manager).

In this scoping review, a critical appraisal of individual sources of evidence has been performed (Table 1).

**Table 1:** Critical appraisal of individual sources of evidence.

First author	Title	Critical appraisal
Goh et al.	Virtual and augmented reality for surgical training and simulation in knee arthroplasty	Few studies using IVR have described the cost considerations explicitly Although available evidence has demonstrated favourable immediate skill acquisition and transfer, current gaps in the literature on IVR pertain to its effectiveness in ensuring long‐term skill retention, skill improvements for experienced surgeons, coordinated training for ancillary operating room staff, as well as the cost‐effectiveness of its incorporation into training programmes Further longitudinal studies are needed to address these questions
Barry et al.	Perioperative outcomes of immersive virtual reality as adjunct anaesthesia in primary total hip and knee arthroplasty	The major limitation of the study includes the relatively small sample size, which can result in inadequate power for insignificant findings Patient selection bias is inherent as patients were offered and eventually self‐selected the IVR experience for their surgery Multiple anaesthesia providers and surgeons were involved in the patients’ surgical care episode, and subjective differences in anaesthesia practice and procedural techniques exist. Furthermore, no blinding can be performed given the application of the IVR device, and such ongoing interactions may have also led to differences in the amount of sedation given by the treating anaesthesia providers Baseline and postoperative mental status or health assessments were not obtained, and could further elucidate the benefits of decreased pharmaceutical requirements between the groups. A well‐powered randomised controlled trials will be needed to further delineate the potential benefits of IVR in total joint arthroplasty.
Peuchot et al.	Intraoperative VR distraction in TKA under spinal anaesthesia: A preliminary study	The analysis was limited to TKA for primary osteoarthritis because of the institution experience in this area and to ensure matched groups. Another limitation was the VR material and its lack of autonomy—the headset had turned off in 3 patients due to low battery, stopping the VR distraction. The study was unblinded and can lead to bias There was no randomisation of patients This was a single‐centre study with several surgeons surgeons (5) and anaesthesiologists (4)
Li L	Effect of remote control augmented reality multimedia technology for postoperative rehabilitation of knee joint injury	Due to limited samples and space, this study was not comprehensive and in‐depth enough For example, when the effects of rehabilitation were evaluated, it only analysed the pain relief, swelling and structural and functional recovery. There were no statistics on the enthusiasm of the patients who were interested in training In future study and work, it will expand the sample to further comprehensively and in‐depth study this issue.
Chughtai et al.	The role of virtual rehabilitation in total and unicompartmental knee arthroplasty	The small sample size and the retrospective review may render the study underpowered and introduce selection bias. Additionally, the follow‐up time may be relatively short, which may not accurately represent the true final outcomes in the cohort.
Hadamus et al.	Assessment of the effectiveness of rehabilitation after total knee replacement surgery using sample entropy and classical measures of body balance	The presented study has some limitations—small number of participants Statistical power was in range 0.0561 (for SampEn_ML during trial with eyes closed) up to 0.7901 (for SampEn_AP during trial with eyes open)
Hadamus et al.	Effectiveness of early rehabilitation with exergaming in virtual reality on gait in patients after total knee replacement	The limitations of this study include the lack of a healthy control group The COVID‐19 pandemic had limited the number of subjects in the study and also the follow‐up period with the patients due to limitations from the National Health Service The use of exergaming sessions could also influence patients’ motivation, the intake of painkillers, the functioning of the neuromuscular system or other factors that were not assessed in this study. The results could also be influenced by psychological complaints, that were not assessed
Maharjan et al.	A novel visualisation system of using augmented reality in knee replacement surgery: Enhanced bidirectional maximum correntropy algorithm	The main limitation of this research is in registering and aligning two different cloud points. While registering and aligning between two pairs of set points, overlapping parts and registration outcomes trapped in local minima are not considered, which could decrease the accuracy, produce the alignment error and affect the processing time.
Bennett et al.	Augmented reality navigation can achieve accurate coronal component alignment during total knee arthroplasty	The limitations of this study were its small sample size, lack of a control group and only radiographic, rather than patient‐reported or observed outcomes. Larger comparative studies will be required to further assess the accuracy of the system
Castellarin et al.	Is total knee arthroplasty surgical performance enhanced using augmented reality? A single‐centre study on 76 consecutive patients	This study implies however a main potential limitation: Only two of the three parameters typically used to guide a TKA surgery were involved, not addressing the cut thickness. These two angles are a standard approach as the surgeon can properly orient the prosthesis with this information alone However, it could be interesting for future studies to assess the precision of this technology, as well as when it comes to reaching other desired positioning parameters, such as the mentioned depth of resection
Tsukada et al.	Augmented reality‐aided unicompartmental knee arthroplasty	This study had several limitations. This was a preliminary study in a single‐arm cohort with a small sample size. Although a pilot study was crucial to assess the feasibility of a larger comparative study, it must be noted that this study could not conclusively demonstrate the clinical effectiveness of the AR‐based navigation system. Second, the results lack generalisability because all surgeries were performed by a single surgeon at a single institute, and the surgeon's experience has been shown to affect the clinical and radiographic outcome of UKA.
Robinson et al.	Variability of landmark identification in total knee arthroplasty	The clinical significance of this study is yet to be determined because there are no comparable reports in the literature of morphologic measures made on a digitised specimen in virtual space. There are also no similar morphologic measures reported for a real specimen. Studies are needed to correlate data collected in virtual space with data collected in the clinical setting. A study comparing the selection of landmarks by multiple individuals on a virtual knee and a knee from a cadaver or patient would help to validate the results of this study.
Tsukada et al.	Augmented reality‐based navigation system applied to tibial bone resection in total knee arthroplasty	The materials used in this study were sawbones without soft tissue The accuracy of registration touching bony landmarks through soft tissues was biased by the thickness of the soft tissue (Tsukada and Wakui 2010). This preclinical study cannot provide surgeons with information on whether the skin incision should be extended or not when using AR‐KNEE in the clinical setting. All bone resections were performed by one surgeon in this study. The accuracy of bone resection may depend on the experience of the surgeon (Kazarian et al. 2019). Multi‐surgeon studies will provide more robust external validity. The AR‐KNEE system may have a learning curve like other navigation systems for TKA (Jenny et al. 2008). It is possible that gaining experience with the AR‐ KNEE system would provide more accurate bone resection.
Tsukada et al.	Augmented reality‐assisted femoral bone resection in total knee arthroplasty	This study had several limitations. Although they assessed coronal alignment using a standing long‐leg radiograph in the clinical study, the sagittal alignment was not measured. The sagittal alignment was measured only in the experimental study using Sawbones utilising CT because the institutional review board determined that additional radiation exposure was unethical in a clinical setting The allocation to the two techniques was not randomised in this study. Although there were no significant differences in the demographic data between the AR‐KNEE and conventional intramedullary guide groups, randomly assigning the intervention could eliminate the influence of confounding variables. Finally, as all TKAs were performed by a single surgeon, the findings lack generalisability to the overall population. Randomised controlled studies including multiple surgeons are needed to confirm the clinical effectiveness of the AR‐KNEE system.
Wang et al.	A HoloLens based augmented reality navigation system for minimally invasive total knee arthroplasty	The problems that may be encountered in actual surgery are multifaceted and difficult to predict, and the evaluation of the methods used in this experiment needs further verification in clinical surgery. At the same time, limited by the processing frequency of the image on the computer, the process of collecting the point cloud often takes several minutes to ensure that the density of the point cloud is large enough.
Pokhrel et al.	A novel augmented reality (AR) scheme for knee replacement surgery by considering cutting error accuracy	Instead of using CT data for 2D to 3D image registration, future research needs to use the updated remaining area to surgery for 2D to 3D image registration with real‐time learning of the remaining area for surgery. Furthermore, other stages of the Iterative closet Point included selection, matching and rejection could be refined. This would lead to potentially more accurate samples that can be converted into 3D
Fucentese et al.	A novel augmented reality‐based surgical guidance system for total knee arthroplasty	The direct assessment of ligament elongation might represent a step forward in terms of soft tissue balancing, but more data must be collected to create a reliable baseline, to be used to better interpret the values recorded intra‐operatively and adjust the surgery plan accordingly. Several cadaver tests performed by the authors and other surgeons involved in the development have confirmed the accuracy of the system. However, clinical research data must be collected to confirm the performance of the system and the impact on clinical outcomes.
Huang M, Scharf S, Chan P	Effects of immersive VR therapy on intravenous patient‐controlled sedation during orthopaedic surgery under regional anaesthesia: A randomised controlled trial	Given 36% of patients chose to remove their HMD early, the addition of more engaging content may be needed to demonstrate a potential effect. The nonblinded nature of the study could lead to differences in the way the treating anaesthesiologist administers additional sedation, such as midazolam and fentanyl There was no significant difference observed in total midazolam and fentanyl use between the two groups, however, their effect on PCS requirements is difficult to quantify. Inclusion of patients in both supine and lateral position may have also confounded results, as these two subgroups are likely to have different sedation requirements, with the lateral positioning being more uncomfortable. It should also be noted that almost half of the patients approached for this study declined, preferring to have analgesia and sedation managed by the anaesthesiologist without IVR
Koo et al.	Enhanced reality showing long‐lasting analgesia after total knee arthroplasty: Prospective, randomised clinical trial	Per‐protocol analysis was used instead of intention‐to‐treat analysis because the protocol was violated in 18 patients. The effect size could be overvalued by per‐protocol analysis and the large number of dropouts (18 of 60), which is a high number for a study of this size. The majority (76%) of the patients in the current trial were females. Considering the difference in visual perception between sexes, no subgroup analyses regarding sex may be warranted. Although a significant difference in time within the group over 5 weeks in VAS and active ROM of the knee was approved in the current trial, it could not gain generalisability because its action induced no significant improvement of the functional scales and was restricted to the knee, which the intervention was targeted towards. It could be because the period of intervention was not long enough; hence, a clinical trial is necessary to determine the therapeutic time window and dosage. Another limitation was not checking the degree of neuroplasticity using electro‐diagnostic or imaging evaluation
Shim et al.	Postoperative rehabilitation using a digital healthcare system in patients with total knee arthroplasty: A randomised controlled trial	The current study had several limitations The sample size was small, and the dropout rate (28.5%) was higher than expected There were differences in the way to track exercise between the groups; the digital health‐care system counted the exercise days based on the data uploaded to the online server when participants completed an entire exercise session, while the participants in the CR group were asked to record both exercise completion and number of repetitions using an exercise diary. As a result, the CR group performed exercise more frequently than the DR group did and adherence was assessed more strictly in the DR group. Also, there were more log‐in records than the number of exercise completions in the DR group, which is probably a result of technical errors such as disconnection or detection, even though actual exercises were performed Thus, the difference in exercise amount between groups is due to the limitations of the digital healthcare system used in this study rather than whether actual exercise was performed, which is considered a part that needs improvement. Despite randomisation, the CR group was older but had a lower BMI than the DR group Age and obesity are not only risk factors for knee OA but also prognostic factors after TKA These baseline differences were analysed and adjusted as confounding variables, and the results did not differ from those of unadjusted analysis. Those differences might not affect the outcomes, as there were no preoperative functional differences between groups
Piqueras et al.	Effectiveness of an interactive virtual telerehabilitation system in patients after total knee arthroplasty: a randomised controlled trial	It was impossible to conduct a double‐blind trial; all participants were instructed not to reveal their intervention group to The evaluator who took functional measurements. At the time the trial was performed there was no evidence about telerehabilitation results; therefore, only patients without clinical complications and with good functionality were randomised. This decision was taken for ethical reasons and to minimise the risk of harm
Ayoade et al.	A novel knee rehabilitation system for the home	The results are limited by the small sample size so should be viewed as suggestive rather than conclusive.
Gur et al.	The effect of virtual reality on pain, kinesiophobia and function in total knee arthroplasty patients: A randomised controlled trial	The short duration of treatment and follow‐up in the current study was a limitation of the study. A longer treatment and follow‐up period could have provided a better understanding of the long‐term effects of VR after TKA. Another limitation was the lack of blinding in the study. The blinding method could not be used on the patients due to the nature of VR. Future studies should have long treatment and follow‐up periods to determine the long‐term effects of VR. New studies should be designed to determine the optimal treatment duration and dosage of VR. In addition, the effects of different application types of VR on different patient groups should be investigated in future studies.
Christiansen et al.	Effects of weight‐bearing biofeedback training on functional movement patterns following total knee arthroplasty: a randomised controlled trial	A small sample size and lack of long‐term follow‐up limit the strength of conclusions Larger trials should further examine efficacy of weight‐bearing bio‐feedback training programs following unilateral TKA. A longer intervention time could be used to assess whether increasing the intervention dose produces adaptations in knee extension strength, knee motion and movement performance. The method of biofeedback could be further studied as well. While the use of constant visual feedback in this study is consistent with other interventions for patients with joint arthroplasty, optimal biofeedback schedules and modes have not been identified. Future work should compare various weight‐bearing biofeedback schedules (e.g., constant, random, summary) and modes (e.g., auditory, tactile, verbal). In addition, the stationary balance board used to provide weight‐bearing biofeedback limited the ability to perform highly dynamic training activities, such as gait, which might have limited the effectiveness of the training.
Bettger et al.	Effects of virtual exercise rehabilitation in‐home therapy compared with traditional care after total knee arthroplasty VERITAS, a randomised controlled trial	The present trial had several limitations They chose to specify costs as they would be billed and reimbursed, and they used clinical site documentation and patient‐reported use. They did not include the costs of technology used by the patients and therapists, equipment home installation and removal or patient co‐pays, deductibles, travel and clinic wait time. Tele‐rehabilitation reimbursement varies by state, and estimated total cost savings need local interpretation. A third of eligible patients declined to participate in the study. Further research is needed on the social and behavioural aspects related to accepting virtual PT as an option for therapy The prescribed exercises varied by therapist and patient in both groups. Although they only studied results among four practices in North Carolina, enrolled patients and sites were broadly representative Research with patients from regions with greater socioeconomic diversity would further inform the utility and applicability.
Yoon et al.	Effects of full immersion virtual reality training on balance and knee function in total knee replacement patients: A randomised controlled study	One limitation of our study is that it required a safe environment, because the HMD worn by the participants makes it difficult to observe surrounding objects, increasing the risk of falls In addition, experimenters had to be cautious because immersion can cause cyber sickness They administered the VR program for only two weeks, and it was difficult to verify its effectiveness because of the relatively short intervention duration. Furthermore, because the training program was only performed in a standing position, they believe that later studies should develop VR programs combined with gait training.
Roig‐Casasús et al.	Balance training with a dynamometric platform following total knee replacement: A randomised controlled trial	There are several limitations to this research Participant unavailability for the follow‐up assessment may be a source of bias. Early postoperative outcomes have been reported to be satisfactory; however, it can take from 6 months to 1 year to reach the functional plateau values after a TKA surgery. The lack of this follow‐up information is one of the main limitations of this trial. A post hoc analysis 22 for the primary outcome and the sample size of the trial resulted in a power of 0.83. There is much debate about the use of post hoc analyses, so the estimation should preferably be conducted a priori Higher scores achieved in the open‐eyes. Romberg test might be influenced by the specific platform training practiced by the experimental group, bearing in mind that the control group participants were less familiar with the instrument. By contrast, being somewhat familiar with the platform did not affect the closed‐eyes test results, which could preclude the above assertion.
Fung et al.	Use of Nintendo Wii Fit™ in the rehabilitation of outpatients following total knee replacement: a preliminary randomised controlled trial	Based on the frequency of outpatient TKR admission and the targeted time for study completion, this study aimed to recruit 60 participants. However, following the successful recruitment of 50 participants, there was a change in the model of physiotherapy care offered to TKR outpatients at the study hospital. This change may or may not have affected the integrity of the results, but the investigators decided to cease recruitment at this point and complete the study using the data sets collected from 50 participants. In addition, the patient satisfaction questionnaire chosen for administration in this study was the standard survey used across the outpatient department Administration of a different survey following study completion would not have accurately depicted opinions of study participation, as the interval between discharge and the identification of this limitation differed between participants. It should be mentioned that despite attempts to maintain exclusivity in the activities performed by the control and study groups in this study, the issue of nonstudy care exists, as described by Freedland et al. Finally, according to the power calculations conducted for this study, the sample size of 50 did not provide sufficient power for most of the outcomes assessed.
Fuchs et al.	The influence of early virtual reality intervention on pain, anxiety, and function following primary total knee arthroplasty	One of the limitations was the short‐timed protocol. In other studies, the protocol was for longer minutes and longer hospitalisation The only non‐patient‐reported parameter is the range of motion. This parameter is best tested throughout a longer period of intervention. Another limitation was the collaboration of our patients. Most of them are older patients, and they needed much help in managing the technology, which could add to their total anxiety. Some of the potential recruits refused to participate due to fear from this technology Another limitation of the study is lack of information about how many medications were used by the patients. The postoperative pain management protocol was the same for all patients, but it is possible that some patients chose to take less or more medications. Further studies should explore the use of VR intervention for a longer period during the rehabilitation process, and the use of a more elderly‐friendly technology.
Pournajaf et al.	Effect of balance training using virtual reality‐based serious games in individuals with total knee replacement: A randomised controlled trial	The authors are aware of some limits of this study, such as the exclusive selection of static balance exercises using only a limited number of serious games included in VR rehabilitation system. Another limitation is the lack of participants’ acceptability and satisfaction in the control group. Finally, the concept of adherence could not be generalised owing to the exclusive number of patients who took part in this study.
Jin et al.	Virtual reality intervention in postoperative rehabilitation after total knee arthroplasty: A prospective and randomised controlled clinical trial	‐
Gianola et al.	Effects of early virtual reality‐based rehabilitation in patients with total knee arthroplasty: A randomised controlled trial	Among its limitations are the impossibility to conduct a double‐blind trial due to the nature of the interventions. To avoid detection bias, the participants were instructed not to reveal their treatment group to the outcome assessors. Also, the study focused only on the efficacy of VR‐based rehabilitation but not its cost‐effectiveness, since previous literature documents that VR is an economical, safe and convenient alternative. Proprioception in both groups was measured using the VR rehabilitation system device; the experimental group may have had a slight advantage of a learning effect since they trained on the device, even if task used for training and assessment were different.
Pandya et al.	Virtual reality distraction decreases routine intravenous sedation and procedure‐related pain during preoperative adductor canal catheter insertion: A retrospective study	There are several limitations to this retrospective study. This was designed as a QI project in the context of clinical care and not a randomised clinical trial. Consequently, there was no blinding or an assigned control group, no placebo and no other form of distraction. Given the small scope of this project, the findings should be considered preliminary The results with VR distraction cannot be applied to every peripheral nerve block site; they chose one procedure location and patient population specifically because patient movement during the VR experience was not expected to be problematic. They also used a standardised ultrasound‐guided perineural catheter insertion technique that is regularly practiced at their institution. This would not apply to nerve blocks of the upper extremity or neuraxial procedures during which VR‐induced movement may create a patient safety risk. Their results may also not be reproducible if using a different technique for adductor canal catheter insertion or different perineural catheter equipment. The intervention was dependent on one portable VR device and the availability of a separate practitioner, uninvolved in direct patient care, to provide coaching on the use of VR during preoperative perineural catheter insertion in a block room. These resources may not be available at every institution.
Garcia‐Sanchez et al.	Effectiveness of virtual reality‐based early postoperative rehabilitation after total knee arthroplasty: A systematic review with meta‐analysis of randomised controlled trials	At first, the medium risk of bias present in each study may affect the findings’ generalisation. On the one hand, the impossibility of concealing the allocation can increase the risk of selection bias On the other hand, performance and detection biases are present due to the impossibility of blind participants, therapists, and assessors, respectively. Due to the impossibility of blinding the participants, it is impossible to eliminate the possible placebo effect of VR Based Rehabilitation, and it is important to consider this when the findings are generalised. These risks can under or overestimate our findings, so it is important to have them present when clinical affirmations are made Secondly, another risk to consider is the risk of publication bias. In some meta‐analyses, when trim‐and‐fill calculation showed major variations of 10% with respect to the original pooled effect, the findings could be under or overestimated. Third, the low number of studies included in the review, especially in each meta‐analysis and subgroup analysis, and the level of evidence shown can reduce the generalisation of the findings Finally, the last limitation is related to the fact that not all studies provided data to assess the effect of each variable over time, and they could not assess the difference between non‐immersive and immersive VR Based Rehabilitation due to the lack of comparisons and data.
Blasco et al.	The efficacy of virtual reality tools for total knee replacement rehabilitation: A systematic review	The number of trials included is suitable for synthesising findings, but interventional designs were heterogeneous, with varying periods of implementation and duration, and meta‐analysis was not possible. The quality assessment suggested a high risk of bias mainly for selection and performance criteria. Two of the randomised trials were pilot studies. Some included studies agreed that continued in‐home training is a practical application requiring further study, but none of the trials appraised this possibility. In addition, preoperative intervention was not studied, and therefore, this aspect of therapy needs evaluation. The healthcare costs associated with the use of VR tools still require assessment.
Batailler et al.	Artificial intelligence in knee arthroplasty: Current concept of the available clinical applications	The review is not comprehensive enough to include all the available technologies but has described the basic and current AI applications in knee arthroplasty. The outputs of machine learning and AI analyses are limited by the types and accuracy of available data sets. Systematic biases in clinical data collection affect the recognition or prediction of AI patterns, such as women and racial minorities due to long‐standing under‐representation in clinical trial and patient registry populations. Ethical considerations regarding the ownership and the use of AI data remain unanswered. The robotic platform storing surgeon and patient information sometimes lacks the patient's express consent and is then used for product development. The next challenge will be to ‘close the loop’ using accurate interconnected data sets and predictive monitoring during the different phases of the patient path (before, during and after knee arthroplasty) to help surgeons and health‐care providers in their decision‐making. The goal is not to replace the health‐care providers but to assist the medical decision collaboratively, combining the doctor's experience and the AI‐based tools. The answer is probably collaborative intelligence to adjust the patient management using predictive models and clinical experience, and make the subsequent surgery better for every patient.
Berton et al.	Virtual reality, augmented reality, gamification, and telerehabilitation: Psychological Impact on orthopaedics patients’ rehabilitation	The main limitation of this review is the heterogeneity of included studies that prevented a meta‐analysis of their results There were no standard procedures or protocols, and different equipment were used. Outcomes measurement methods differed between studies, and much data ere qualitative rather than quantitative Only nine RCTs and 15 nonrandomised studies were analysed. Nevertheless, RCTs were of high or moderate quality and most of the nonrandomised studies had a low risk of bias. The literature lacks data regarding patient's perception of new technology and adherence to therapy. Future research needs to develop specific and objective methods to evaluate the clinical quality of new technologies.
Su et al.	The effectiveness of virtual reality, augmented reality, and mixed reality rehabilitation in total knee arthroplasty: A systematic review and meta‐analysis	There are several potential limitations of our review. The study protocols varied, including the duration and frequency of rehabilitation, and the definition of XR‐based rehabilitation. Also, the sample size of the study is small, there still needs more high‐quality RCTs to be conducted. In addition, some studies did not contain enough information for pooled analysis. Despite their attempts to contact the authors for raw data, they were unable to do so. Furthermore, the language bias was inevitable because all included studies were published in English.
Youssef et al.	Is virtual reality effective in orthopaedics rehabilitation? A systematic review and meta‐analysis	It is important to note that a few limitations exist: Only articles in the English language were included. Studies that assessed psychosocial outcomes were excluded. The scale of quality assessment used, despite being valid and reliable, also did not consider a few risks of bias, such as allocation and reporting bias. All investigated orthopaedic disorders had only a few numbers of eligible studies, which limits this study's ability to reach and present a solid conclusion.
Wang et al.	Technology‐assisted rehabilitation following total knee or hip replacement for people with osteoarthritis: A systematic review and meta‐analysis	There are several limitations to the review. Many studies did not perform a priori sample size calculations, which can increase the risk of underpowered (false‐negative) results. Second, the trials used varied outcome measures, which limited the pooling of results. Consensus on a set of suitable outcome measures needs to be reached for future trials. Furthermore, there is insufficient long‐term follow‐up for ensuring the prolonged effects or safety. A common risk of bias of the studies is a lack of blinding. As blinding of participants and therapists is not possible for most pragmatic trials, including those of technology‐based rehabilitation interventions, future research should pay attention to the methodological aspects to minimise the biases.
Byra et al.	The effectiveness of virtual reality rehabilitation in patients with knee and hip osteoarthritis	The most common type of bias was due to the lack of participants and therapists blinding (in all studies). In most physiotherapy research, blinding is difficult due to the specifics of carrying out interventions. However, blinding all test assessors is possible and should be used in RCT, which concerned about half of the included studies. Another limitation was the lack of the intention‐to‐treat analysis, which concerned eight out of 10 included RCTs. The main limitations of the included trials were the small sample size and the lack of long‐term follow‐up.
Peng et al.	Virtual reality‐based rehabilitation in patients following total knee arthroplasty: A systematic review and meta‐analysis of randomised controlled trials	The study was not without limitations The study protocols differed, including the intervention and control designs, the duration and frequency of rehabilitation and the definition of VR‐based rehabilitation. This may also contribute to the heterogeneity in the study. Although all the studies were RCTs, it was difficult to blind patients and therapists during rehabilitation, which may inevitably bias the self‐reported outcomes. Owing to the limited sample size, the advantage of VR‐based rehabilitation in TKA still needs more high‐quality RCTs to be conducted.
Gazendam et al.	Virtual reality rehabilitation following total knee arthroplasty: A systematic review and meta‐analysis of randomised controlled trials	This review is limited by the current available literature. There were significant variations in both the VR and standard protocols with respect to type of exercises and duration of rehabilitation. This variability makes it more difficult to make meaningful conclusions from synthesised data in a meta‐analysis. Given that no trials have analysed different VR‐rehabilitation protocols, the optimal strategy and protocol remains unknown Additionally, they rated the quality of evidence as moderate to very low, indicating that current evidence is inadequate to allow a clear conclusion. Small sample size in the meta‐analysis is one of the main concerns.
Iacono et al.	The use of augmented reality for limb and component alignment in total knee arthroplasty: systematic review of the literature and clinical pilot study	The present study had several limitations. They cannot assess the accuracy of Knee + augmented reality system because all surgeries were carried out by an experienced surgeon. It is known that the accuracy of bone resection depends on the experience of the surgeon. Moreover, the present research consisted of a clinical pilot study characterised by a limited sample size. Analysing the results of the review, it seems clear that the use of AR in knee arthroplasty is still in its infancy. This contributes to the difficulties in assessing its reliability and, more importantly, its accuracy. The translation from pilot study to high‐level prospective studies is warranted to assess accuracy, limitations and cost‐effective analysis compared to conventional techniques.
Daniel et al.	Augmented reality for assistance of total knee replacement	With the aim of improving the performance of the systems it is necessary to do some changes in the characteristics of the capture devices, like use of reflective markers instead of common markers, as well as a camera that allows detecting infrared frequency band of the light spectrum, improving the accuracy in the detection of the markers without depending on the conditions of light; in addition, the use of more markers is proposed to have different points of view and references for the location of the virtual models. The changes of the lighting in the scene generate noise in the identification of the marker, which may compromise the performance of the system during a surgery Therefore, in future research the use of reflective markers and infrared cameras is proposed, as well as a greater set of markers to increase the accuracy of the system, developing a system that does not depend on a single reference point.
Edwards et al.	Immersive virtual reality enables technical skill acquisition for scrub nurses in complex revision total knee arthroplasty	This study has several limitations It was a single‐arm study with small numbers and a relatively large loss to follow‐up. The cohort examined was specific being orthopaedic scrub nurses and they only focused on one operation, which may limit the generalisability of their results to other cohorts or operations. Whilst there was no control group, the participants acted as their own controls, having had a baseline assessment prior to the training. Given that the study took place over 4 weeks, they cannot guarantee that the training effect observed was entirely attributed to the iVR training. Although no participants scrubbed for a revision knee procedure during the study period, they may have sought out other training materials to aid their performance, such as the surgical technique manual. This may have biased their results Third, they could not assess performance in the real‐world operating theatre or link their performance to any patient outcomes. However, the physical world assessment used was designed to reflect the tasks required during a real operation and the real equipment was used. Another limitation was the analysis of the iVR videos for errors and prompts was not determined automatically by the computer software. Despite excellent interobserver reliability for these measurements, they did not examine repeatability. If performed, this may have strengthened the validity of this data. Finally, some of the participants were still improving in the final training session and had not plateaued on the learning curve. Participants received four sessions based on previous iVR studies in hip arthroplasty showing plateau of skills in surgeons. Studies of novices learning simulated arthroscopy have suggested five to six sessions are required before plateau of technical skills. Based on the present study, it is unknown whether more sessions would have benefited scrub nurse participants learning rTKA. This combined with the lack of expert benchmarking, means they cannot comment on how many training sessions are sufficient to reach expert levels of proficiency.
Vestermark et al.	Cognitive training for robotic arm‐assisted unicompartmental knee arthroplasty through a surgical simulation mobile application	This randomised comparative study is not without limitation. They acknowledge the overall underpowered small sample size but prioritised an inclusion of users at different stages of their surgical training While the ‘study session’ had a designated amount of time for preparation, there was an inherent variation in the amount of time spent studying, which was dependent on uncontrolled factors such as participant motivation. They recognise that the paper‐based reference guide is not a formally validated learning tool. It is possible that the outcome difference may be because of a poorly designed technique guide more so than an intuitive surgical simulation app. Additionally, the 3‐week retention test was not proctored, allowing for the potential use of extraneous resources. It bears noting that the Touch Surgery app does not evaluate manual dexterity or surgical technique. The primary outcomes assessed were posttest performance and information retention, and therefore, they cannot conclude whether this translates to improved surgical skill set in the operating room.
Newman et al.	Content and face validity assessment of the Sim‐K Haptic‐feedback enhanced total knee replacement virtual reality simulator	With respect to limitations of this study, the sample size of 30 is relatively small and the inclusion of only male subjects is a potential source of bias, however comparable studies have used similar or smaller size samples 5 and the gender distribution was not by design, but is reflective of the staffing within the local orthopaedic department.
Hall et al.	Technology‐enhanced learning in orthopaedics: Virtual reality and multi‐modality educational workshops may be effective in the training of surgeons and operating department staff	There are limitations to the current study. First, there was no comparison between performance in the educational activities and the real‐world setting. This comparison would require multiple workplace‐based assessments for each participant in surgical and nonsurgical clinical environments before and after the training interventions, which was not possible due to the significant disruption to routine elective services. Second, the design of the interventions in this study was informed by a trainee focus group session, which is likely to have increased trainee buy‐in and ensured the training was mapped to their needs.
McKinney et al.	Virtual reality training in unicompartmental knee arthroplasty: a randomised, blinded trial	There were several limitations to the study. One limitation is the use of residents from all training years. However, an implant system not previously used at this centre was chosen for the study, and residents were randomised within their training year to obtain a more even distribution in each training group. Another limitation is the use of only one evaluator. Assessment was strictly a procedural assessment, and no knowledge‐based test was performed. Therefore, comprehension of the procedure beyond the correct completion of surgical steps was not evaluated. Though the use of SawBones models is common in orthopaedic training, the use of SawBones instead of cadaver models may be considered a limitation as well. The study also does not address long‐term retention of the procedure over time nor does it evaluate the transfer of this learning method to live surgery beyond the SawBones model. This too is an area of future study
Alpaugh et al.	Immersive technologies for total knee arthroplasty surgical education	As is the case with all emerging technologies, there are potential drawbacks to their use. VR and AR educational tools should create generalisable learning environments that promote transfer skills into the real world. Unfortunately, it is unclear if these technologies can be a substitute for live hands‐on surgical experience when it comes to motor execution and tactile feedback. On the other hand, the number of instruments used and their complexity in orthopaedic surgery is rising. A surgeon might benefit from preprocedural VR/AR training with specific instrumentation to become more facile with an instrument system before using it for the first time in a live surgery. Cost is a factor that must be considered by acquiring immersive technology. Although immersive technology has the potential to be cost‐effective, a recent systematic review of VR platforms for surgical training in orthopaedic surgery stresses that further work must be done to validate simulated training platforms to justify financial investment in these products.
Mandal et al.	Surgery training and simulation using virtual and augmented reality for knee arthroplasty	The intraoperative usage of HMD and their function in clinical treatment have not yet been thoroughly proven, despite the popularity of AR for surgical navigation. If larger clinical studies show benefits over present practice, HMDs may shortly be used to exhibit critical data, photos and even fluoroscopy imaging in the surgeon's visual field. More sophisticated tracking software needs to be developed to maximise the usefulness of these devices within the operating theatre. The solitary solution is electromagnetic surveillance, which does not require a line of vision, although the techniques are frequently limited by interference from metallic instruments
Shaikh et al.	Exposure to XR and artificial intelligence‐based manifestations: A primer on the future of hip and knee arthroplasty	This XR review has potential limitations The largest consideration is that the marketplace and literature remain immature. The studies reviewed were often pilots consisting of small cohorts. As with any new technology, further investigation with long‐term, comparative assessment is necessary to ascertain clinical relevance. Currently, it is unclear whether the improved precision and accuracy provided by XR during knee and hip arthroplasty procedures will result in improved long‐term functional outcomes. Nevertheless, the increased accuracy provided by the combination of AI and XR technology may mitigate the limited shortcomings of hip and knee arthroplasty. The limitations of XR technology itself are important to discuss, as this lends to the limitations of the review. Digital content viewed within XR settings are subject to temporal errors given the requirement for live updates arising from the dynamic motion between the user and object of interest. Image quality depends on the state of added systems, such as inertial sensors, input data, tracking sensor technology and registration. Although various motion prediction techniques are used for latency compensation, these temporal considerations have performance implications.
Bagaria et al.	Robotic‐assisted knee arthroplasty (RAKA): The technique, the technology and the transition	Although today there is limited data demonstrating the superiority of robotic platforms, there is clear evidence that these platforms enable surgeons to position a component with greater precision in a position that he desires While each surgeon may have his own ‘ideal positioning and concept of alignment’ and they may keep changing with time, the ability to achieve the desired position is enhanced

Abbreviations: AI, artificial intelligence; CR, conventional rehabilitation; CT, computed tomography; DR, digital healthcare rehabilitation; HMD, head mounted display; IVR, immersive virtual reality; OA, osteoarthritis; PCS, patient controlled sedation; PT, physiotherapy; ROM, range of motion; TKA, total knee arthroplasty; TKR, total knee replacement; UKA, unicompartmental knee arthroplasty; VAS, visual analogue scale; VR, virtual reality; XR, extended reality.

The final search strategy for PubMed was as follows:

(Search 1) (“virtual reality”[MeSH Terms] OR (“virtual”[All Fields] AND “reality”[All Fields]) OR “virtual reality”[All Fields]) AND (“arthroplasty, replacement, knee”[MeSH Terms] OR (“arthroplasty”[All Fields] AND “replacement”[All Fields] AND “knee”[All Fields]) OR “knee replacement arthroplasty”[All Fi elds] OR (“knee”[All Fields] AND “arthroplasty”[All Fields]) OR “knee arthroplasty”[All Fields])

(Search 2) (“augmented reality”[MeSH Terms] OR (“augmented”[All Fields] AND “reality”[All Fields]) OR “augmented reality”[All Fields]) AND (“arthroplasty, replacement, knee”[MeSH Terms] OR (“arthroplasty”[All Fields] AND “replacement”[All Fields] AND “knee”[All Fields]) OR “knee replacement arthroplasty”[All Fields] OR (“knee”[All Fields] AND “arthroplasty”[All Fields]) OR “knee arthroplasty”[All Fields])

(Search 3) (“augmented reality”[MeSH Terms] OR (“augmented”[All Fields] AND “reality”[All Fields]) OR “augmented reality”[All Fields] OR (“mixed”[All Fields] AND “reality”[All Fields]) OR “mixed reality”[All Fields]) AND (“arthroplasty, replacement, knee”[MeSH Terms] OR (“arthroplasty”[All Fields] AND “replacement”[All Fields] AND “knee”[All Fields]) OR “knee replacement arthroplasty”[All Fields] OR (“knee”[All Fields] AND “arthroplasty”[All Fields]) OR “knee arthroplasty”[All Fields])

Appendix A contains the final search strategy for Cochrane Central Register for Controlled Trials and Google Scholar.

Data extracted included article characteristics (e.g., first author, title, year of publication), type of technology (e.g., XR, and/or VR and/or AR and/or MR), the type and number of subjects (e.g., knee models/TKA patients for cohort studies/RCT/experimental study, or number of studies included for systematic review and/or meta‐analysis), the theme of application for the type of technology (e.g., clinical practice/education/research), the subtheme under each theme, the kind of article and also the main conclusion from each article.

The relevant articles from the literature review were categorised into three main application themes for knee arthroplasty: clinical practice, education and research. They were further classified based on the subthemes under each main theme.

We employed the ‘snowballing’ technique and included articles from systematic reviews that our literature search missed.

## RESULTS

**Figure 2:**
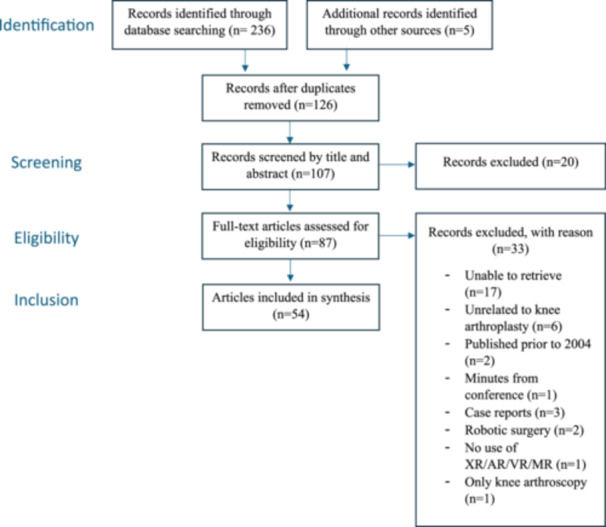
PRISMA methods flow chart. AR, augmented reality; MR, mixed reality; PRISMA, preferred reporting items for systematic reviews and meta‐analysis; VR, virtual reality; XR, extended reality.

A total of 236 papers were identified across the electronic databases and 5 were identified from the reference list of subsequently included articles. Of these articles, 115 were duplicates and were removed. After screening by title and abstract, 20 articles were not eligible for full‐text review, with 87 full‐text articles to be retrieved and assessed for eligibility. Of these, 33 articles were excluded for the following reasons: 17 were unable to be retrieved, 6 were unrelated to knee arthroplasty, 2 were published before 2004, 1 was minutes from a conference, 3 were case reports, 2 included robotic surgery solely without integration with XR, VR, AR or MR, 1 had no use of XR, VR, AR, MR, 1 included only knee arthroscopy without arthroplasty. The remaining 54 articles were considered eligible for this review (Figure 2).

Table 1 summarises the characteristics of the articles, together with the year of publication, type of technology used, type and number of subjects, theme and subtheme of technology application, type of article and conclusion.

The articles were classified into 3 themes—‘Clinical Practice’, ‘Education’ and ‘Research’. These 3 themes were further classified into subthemes as follows:

1. Clinical practice
  a. Rehabilitation
  b. Surgery
  c. Anaesthesia
2. Education
  a. Surgeon
  b. Nurses
3. Research
  a. Artificial intelligence
  b. Robotic assisted surgery

The relevant data for each source of evidence is summarised in Table 2.

**Table 2:** Summary of characteristics of the articles, year of publication, type of technology used, type and number of subjects, theme and subtheme of technology application, study design and conclusions.

First author	Title	Sample size	Characteristic of technology	Theme	Type of technology	Sub‐theme	Year	Study design	Conclusion
Goh et al.	Virtual and augmented reality for surgical training and simulation in knee arthroplasty	‐	Precision OS technology as an immersive, multisensory immersive VR training environment	Clinical practice education	Augmented reality mixed reality virtual reality extended reality	Surgery surgeons	2021	Systematic review and meta‐analysis case example	XR technologies for orthopaedic training and practice have shown great promise by enabling the surgeon to visualise patient‐specific anatomy in real‐time, enhancing preoperative planning and providing intraoperative guidance to improve the precision of surgical interventions. Most importantly, IVR technology has demonstrated its potential to revolutionise modern surgical training and optimise surgical performance in a cost‐efficient manner, ultimately leading to improved patient outcomes.
Barry et al.	Perioperative outcomes of immersive virtual reality as adjunct anaesthesia in primary total hip and knee arthroplasty	Total knee arthroplasty patients (*n *= 10)	Immersive VR as an adjunct to spinal anaesthesia	Clinical practice	Virtual reality	Anaesthesia	2022	Cohort study	Use of spinal anaesthesia with immersive VR to perform TKA appears to be associated with less intraoperative sedative medication usage than spinal anaesthesia alone
Peuchot et al.	Intraoperative virtual reality distraction in TKA under spinal anaesthesia: A preliminary study	Total knee arthroplasty patients (*n *= 20)	Validated VR headset (HyponVR) during total knee arthroplasty	Clinical practice	Virtual reality	Anaesthesia	2021	Cohort study	VR as immersive distraction led to no difference in patient anxiety post TKA or patient satisfaction, but led to decreased intra‐operative adverse events and increased post operative comfort
Li L	Effect of remote control augmented reality multimedia technology for postoperative rehabilitation of knee joint injury	Total knee arthroplasty patients (*n *= 40)	AR‐based rehabilitation training system— real scene training data acquisition, virtual scene construction, virtual and real fusion	Clinical practice	Augmented reality	Rehabilitation	2022	Cohort study	Compared with traditional methods, the rehabilitation training method based on AR showed strong advantages in alleviating postoperative pain and helping structural and functional recovery.
Chughtai et al.	The role of virtual rehabilitation in total and unicompartmental knee arthroplasty	Total knee arthroplasty patients (*n *= 18). Unicompartmental Knee arthroplasty patients (*n *= 139)	Virtual exercise rehabilitation assistant (VERA)—Telerehabilitation system with instruction avatar, 3D motion measurement and analysis software and real‐time televise capability	Clinical practice	Virtual reality	Rehabilitation	2019	Cohort study	The VERA platform used in this study both enables on‐demand rehabilitation sessions for the patients, while also encouraging clinician–patient interaction beyond the hospital setting, and offers the advantage of cost savings, convenience, at‐home monitoring, and coordination of care, all of which are geared to improve adherence and overall patient satisfaction
Hadamus et al.	Assessment of the effectiveness of rehabilitation after total knee replacement surgery using sample entropy and classical measures of body balance	Total knee arthroplasty patients (*n *= 42).	VR‐based training on balance parameters in patients undergoing rehabilitation shortly after total knee replacement surgery	Clinical practice	Virtual reality	Rehabilitation	2021	Cohort study	This study aimed to assess the effect of adjunct VR‐based training on balance parameters in patients undergoing rehabilitation shortly after total knee replacement surgery. Our results indicate that, unfortunately, no significant improvement in the postural stability parameters assessed was noted either following a standard rehabilitation protocol or an enhanced protocol with VR training
Hadamus et al.	Effectiveness of early rehabilitation with exergaming in virtual reality on gait in patients after total knee replacement	Total knee arthroplasty patients (*n *= 59)	Standard rehabilitation program with additional exercises in nonimmersive VR in improving spatiotemporal and pressure distribution gait parameters	Clinical practice	Virtual reality	Rehabilitation	2022	Cohort study	Additional exercises in VR do not significantly improve pressure and spatiotemporal gait parameters compared with standard rehabilitation alone.
Maharjan et al.	A novel visualisation system of using augmented reality in knee replacement surgery: Enhanced bidirectional maximum correntropy algorithm	Total knee arthroplasty patients (*n *= 17)	AR visualisation system in TKA (Enhanced bidirectional maximum correntropy algorithm)	Clinical practice	Augmented reality	Surgery	2020	Cohort study	The proposed system was reliable and favourable which helps in eliminating alignment error by ascertaining the optimal rigid transformation between two cloud points and removing the outliers and non‐Gaussian noise The proposed AR system helps in accurate visualisation and navigation of anatomy of knee such as femur, tibia, cartilage, blood vessels and so forth.
Bennett et al.	Augmented reality navigation can achieve accurate coronal component alignment during total knee arthroplasty	Total knee arthroplasty patients (*n *= 18)	Total knee arthroplasty utilising a Knee+ AR system to achieve accurate coronal component alignment	Clinical practice	Augmented reality	Surgery	2023	Cohort study	Augmented reality navigation can achieve accurate alignment of total knee arthroplasty with a low rate of component malposition in the coronal plane Acceptable and consistent accuracy can be achieved from the initial adoption of this technique, however, some sagittal outliers were identified and there is a clear learning curve with respect to operating time
Castellarin et al.	Is total knee arthroplasty surgical performance enhanced using augmented reality? A single‐centre study on 76 consecutive patients	Total knee arthroplasty patients (*n *= 76)	Smart augmented reality glasses were used to provide real‐time information on the planned inclination of the cuts	Clinical practice	Augmented reality	Surgery	2024	Cohort study	AR‐based system achieved varus and slope cut angles with errors less than 1 degree when matched with the preplanned configuration. There was minimal impact on surgery duration and relative patient bleeding.
Tsukada et al.	Augmented reality‐aided unicompartmental knee arthroplasty	Unicompartmental knee arthroplasty patients (*n *= 11)	AR‐based portable navigation system for proximal tibial resection during UKA	Clinical practice	Augmented reality	Surgery	2022	Cohort study	This study could not conclusively demonstrate the clinical effectiveness of the AR‐based navigation system. The results lack generalisability because all surgeries were performed by a single surgeon at a single institute and surgeon's experience has been shown to affect the clinical and radiographic outcome of UKA
Robinson et al.	Variability of landmark identification in total knee arthroplasty	55 participants	Using 3D glasses (CrystalEyes, StereoGraphics, Beverly Hills, CA) to identify four anatomical landmarks in distal femur	Clinical practice	Virtual reality	Surgery	2006	Cohort study	Certain anatomic landmarks used in total knee arthroplasty are not reliable when tested in VR
Tsukada et al.	Augmented reality‐based navigation system applied to tibial bone resection in total knee arthroplasty	Knee models	AR‐based navigation system (AR‐KNEE) in tibial bone resection during total knee arthroplasty	Clinical practice	Augmented reality	Surgery	2019	Experimental study	This pilot study using sawbones suggested that the AR‐KNEE system may provide reliable accuracy for coronal, sagittal, and rotational alignment in tibial bone resection during total knee arthroplasty
Tsukada et al.	Augmented reality‐assisted femoral bone resection in total knee arthroplasty	Knee models (*n *= 10) total knee arthroplasty patients (*n *= 72)	AR‐based navigation system (AR‐KNEE) in measuring resection angle of the distal femur in total knee arthroplasty	Clinical practice	Augmented reality	Surgery	2021	Experimental study	The AR‐based navigation system may enable surgeons to perform distal femoral resection more accurately than with the conventional intramedullary guide during TKA
Wang et al.	A HoloLens based augmented reality navigation system for minimally invasive total knee arthroplasty	Knee models	Microsoft Hololens (AR navigation system) for MIS‐TKA	Clinical practice	Augmented reality	Surgery	2019	Mechanistic study	Microsoft Hololens for MIS‐TKA can provide the surgeon with accurate location information when the field of view is narrow. The results have shown the potential use of the HoloLens to provide minimally invasive TKA with real‐time intuitive surgical visualisation without introducing X‐ray radiation
Pokhrel et al.	A novel augmented reality (AR) scheme for knee replacement surgery by considering cutting error accuracy	Knee models	AR navigation scheme for knee arthroplasty	Clinical practice	Augmented reality	Surgery	2019	Mechanistic study	The proposed AR navigation system reduces the processing time and increases accuracy intraoperatively
Fucentese et al.	A novel augmented reality‐based surgical guidance system for total knee arthroplasty	Knee models	AR surgical guidance system for TKA (NextAR TKA)	Clinical practice	Augmented reality	Surgery	2021	Mechanistic study	The use of smart glasses and integrated sensors improves the efficiency of the procedure, particularly when coupled with single‐use instrumentation. A novel protocol for soft tissue assessment allows for a 3‐dimensional evaluation of the ligaments and a better measurement of the effect of tibial rotation
Huang M, Scharf S, Chan P	Effects of immersive virtual reality therapy on intravenous patient‐controlled sedation during orthopaedic surgery under regional anaesthesia: A randomised controlled trial	Total knee arthroplasty patients (*n *= 30)	Immersive VR	Clinical practice	Virtual reality	Anaesthesia	2020	Randomised controlled trial	There was no significant difference in patient‐controlled intra‐operative propofol use during joint replacement surgery under regional anaesthesia when using IVR compared with the control group receiving no IVR, reflected in the total dose required per case, the amount per hour, and the amount administered in any hour.
Koo et al.	Enhanced reality showing long‐lasting analgesia after total knee arthroplasty: Prospective, randomised clinical trial	Total knee arthroplasty patients (*n *= 60)	Using real‐time embodiment and virtual limb presence as VR‐mediated analgesia	Clinical practice	Virtual reality	Anaesthesia	2018	Randomised controlled trial	Enhanced reality might induce long‐lasting analgesia through more potentiated brain plasticity and improve active range of motion
Shim et al.	Postoperative rehabilitation using a digital healthcare system in patients with total knee arthroplasty: A randomised controlled trial	Total knee arthroplasty patients (*n *= 56)	Augmented reality‐based digital healthcare system for home exercises	Clinical practice	Augmented reality	Rehabilitation	2023	Randomised controlled trial	The use of a digital healthcare system based on AR was associated with higher satisfaction and improved the functional outcomes, pain, and quality of life of patients after TKA
Piqueras et al.	Effectiveness of an interactive virtual telerehabilitation system in patients after total knee arthroplasty: A randomised controlled trial	Total knee arthroplasty patients (*n *= 142)	Interactive virtual telerehabilitation with wireless sensors, an interactive patient application and web portal for therapist to receive data	Clinical practice	Virtual reality	Rehabilitation	2013	Randomised controlled trial	2‐week interactive virtual telerehabilitation programme is at least as effective as conventional therapy. Telerehabilitation is a promising alternative to traditional face‐to‐face therapies after discharge from total knee arthroplasty. Offers the advantage of decreasing the number of in‐person sessions at the rehabilitation centre and associated use of costly medical transport
Ayoade et al.	A novel knee rehabilitation system for the home	Total knee arthroplasty patients (*n *= 15)	Rehabilitation visualisation interface with two inertial motion sensor and a low‐fidelity animation on a laptop for rehabilitation exercises	Clinical practice	Virtual reality	Rehabilitation	2014	Randomised controlled trial	The rehabilitation visualisation system is easy to use by seniors unsupervised, effective in ensuring correct performance of home knee exercises, motivates patients at early rehabilitation phase, facilitates communication of rehabilitation progress with therapists, improves knee range of motion and accelerates functional recovery, and improves mental health of patients
Gur et al.	The effect of virtual reality on pain, kinesiophobia and function in total knee arthroplasty patients: A randomised controlled trial	Total knee arthroplasty patients (*n *= 21)	Immersive VR application (BOBOVR Z5 model VR devices)	Clinical practice	Virtual reality	Rehabilitation	2023	Randomised controlled trial	In the early period following total knee arthroplasty, using virtual reality integrated with exercise helps to reduce pain, kinesiophobia and pain catastrophising, and to improve functional outcomes in female patients. However, VR did not contribute to the improvement of the quality of life.
Christiansen et al.	Effects of weight‐bearing biofeedback training on functional movement patterns following total knee arthroplasty: A randomised controlled trial	Total knee arthroplasty patients (*n *= 26)	Standard‐of‐care rehabilitation augmented with weight‐bearing biofeedback training using the Nintendo Wii Fit Plus game and Wii Balance Board	Clinical practice	Virtual reality	Rehabilitation	2015	Randomised controlled trial	The addition of a 6‐week intervention of weight‐bearing biofeedback training to standard rehabilitation post‐TKA did not improve functional weight‐bearing symmetry However, weight‐bearing biofeedback training successfully increased knee extension moments during walking soon after TKA
Bettger et al.	Effects of virtual exercise rehabilitation in‐home therapy compared with traditional care after total knee arthroplasty VERITAS, a randomised controlled trial	Total knee arthroplasty patients (*n *= 287)	Virtual physical therapy program with an avatar coach, 3D biometric and skilled telerehabilitation	Clinical practice	Virtual reality	Rehabilitation	2019	Randomised controlled trial	Relative to traditional home or clinic PT, virtual PT with telerehabilitation for skilled clinical oversight significantly lowered 3‐month health‐care costs after TKA while providing similar effectiveness.
Yoon et al.	Effects of full immersion virtual reality training on balance and knee function in total knee replacement patients: A randomised controlled study	Total knee arthroplasty patients (*n *= 30)	Full immersion VR training program	Clinical practice	Virtual reality	Rehabilitation	2020	Randomised controlled trial	VR training produced better early balance ability and knee function than what was seen in the control group
Roig‐Casasús et al.	Balance training with a dynamometric platform following total knee replacement: A randomised controlled trial	Total knee arthroplasty patients (*n *= 43)	Additional balance training with dynamometric platform consisting of tests related to stability challenges, weight shifting and moving to the limits of stability	Clinical practice	Virtual reality	Rehabilitation	2017	Randomised controlled trial	Participants with TKR who have followed a 4‐week training program using a dynamometric platform improved balance performance to a higher extent than a control group training without such a device
Fung et al.	Use of Nintendo Wii Fit™ in the rehabilitation of outpatients following total knee replacement: A preliminary randomised controlled trial	Total knee arthroplasty patients (*n *= 50)	Physiotherapy session followed by 15 min of Wii Fit gaming activities	Clinical practice	Virtual reality	Rehabilitation	2012	Randomised controlled trial	Overall outcomes for TKR patients that undertook Wii Fit exercises did not differ from those who undertook physiotherapy exercises, indicating that Wii Fit can be applied as an adjunct intervention without risk of poorer outcomes compared to traditional physiotherapy exercises.
Fuchs et al.	The influence of early virtual reality intervention on pain, anxiety and function following primary total knee arthroplasty	Total knee arthroplasty patients (*n *= 55)	Using the Samsung Gear VR for continuous passive motion device for 15 min	Clinical practice	Virtual reality	Rehabilitation	2022	Randomised controlled trial	Virtual reality intervention in the immediate postoperative period following total knee arthroplasty decrease pain and anxiety but did not influence the pain, anxiety and long‐term function results more than conventional physiotherapy
Pournajaf et al.	Effect of balance training using virtual reality‐based serious games in individuals with total knee replacement: A randomised controlled trial	Total knee arthroplasty patients (*n *= 56)	Nonimmersive VR‐based serious games with biofeedback on clinical, gait and postural outcomes in patients with unilateral total knee arthroplasty	Clinical practice	Virtual reality	Rehabilitation	2022	Randomised controlled trial	Balance training via nonimmersive VR‐based serious games is effective but not superior to conventional therapy after TKR
Jin et al.	Virtual reality intervention in postoperative rehabilitation after total knee arthroplasty: A prospective and randomised controlled clinical trial	Total knee arthroplasty patients (*n *= 66)	VR (Mide Technology Inc.) intervention through asking patients to row a boat using knee flexion in an immersive virtual environment	Clinical practice	Virtual reality	Rehabilitation	2018	Randomised controlled trial	Clinical application of VR intervention can aid rehabilitation, reduce postoperative pain, and improve functional recovery in OA patients undergoing TKA
Gianola et al.	Effects of early virtual reality‐based rehabilitation in patients with total knee arthroplasty: A Randomised Controlled Trial	Total knee arthroplasty patients (*n *= 85)	Early rehabilitation with VR via the Virtual Reality Rehabilitation System (VRRS) after total knee arthroplasty	Clinical practice	Virtual reality	Rehabilitation	2020	Randomised controlled trial	Early inpatient VR‐based rehabilitation is not superior to traditional rehabilitation in relieving pain and improving their functional outcomes, whereas it enhanced proprioception in these TKA patients
Pandya et al.	Virtual reality distraction decreases routine intravenous sedation and procedure‐related pain during preoperative adductor canal catheter insertion: A retrospective study	Total knee arthroplasty patients (*n *= 14)	VR distraction (Hypervision 2D Virtual Reality Glasses) with intravenous sedation upon request	Clinical practice	Virtual reality	Anaesthesia	2017	Retrospective study	VR distraction during preoperative adductor canal catheter insertion decreased the use of intravenous opioid and sedatives and reduced procedure‐related pain without increasing the procedural duration. VR distraction may provide an effective nonpharmacological alternative to intravenous sedation for the ultrasound‐guided placement of certain perineural catheters
Garcia‐Sanchez et al.	Effectiveness of virtual reality‐based early postoperative rehabilitation after total knee arthroplasty: A systematic review with meta‐analysis of randomised controlled trials	12 studies 997 patients (*n *= 997)	Immersive and non‐immersive VR‐ based rehabilitation after total knee arthroplasty	Clinical practice	Virtual reality	Rehabilitation	2023	Systematic review	Virtual reality‐based rehabilitations is an effective therapy to improve knee pain, knee function, dynamic balance, knee flexion ROM and extension strength, in comparison to conventional therapy, in patients after TKA. In addition, the effect of VR based rehabilitation is maintained over 3 or 6 months on knee pain and function.
Blasco et al.	The efficacy of virtual reality tools for total knee replacement rehabilitation: A systematic review	6 studies	Multi‐modal intervention with VR tools used to augment conventional rehabilitation—Nintendo Wii and Wii Balance Board most frequently used device	Clinical practice	Virtual reality	Rehabilitation	2021	Systematic review	Physical therapy augmented with VRT and alternative VRT training had no advantage over conventional rehabilitation. Therapy with and without VRT were similarly effective in improving functional outcomes and resolving pain
Batailler et al.	Artificial intelligence in knee arthroplasty: Current concept of the available clinical applications	132 studies	AI‐based tools	Clinical practice	Augmented reality mixed reality virtual reality	Surgery	2022	Systematic review	AI‐based tools improve the decision‐making process, surgical planning, accuracy and repeatability of surgical procedures.
Berton et al.	Virtual reality, augmented reality, gamification, and telerehabilitation: Psychological impact on orthopaedics patients’ rehabilitation	24 studies 2472 patients—including gamification and telerehabilitation for hip and knee arthroplasty patients	AR, VR, gamification or telerehabilitation for both TKA, UKA, TKR	Clinical practice	Augmented reality virtual reality	Rehabilitation	2020	Systematic review and meta‐analysis	Heterogeneity of studies prevented a meta‐analysis of results. Remote virtual technologies allow the delivery of high‐quality care at reduced costs
Su et al.	The effectiveness of virtual reality, augmented reality, and mixed reality rehabilitation in total knee arthroplasty: A systematic review and meta‐analysis	14 studies 989 patients	XR postoperative rehabilitation	Clinical practice	Augmented reality virtual reality extended reality	Rehabilitation	2024	Systematic review and meta‐analysis	XR‐based rehabilitation improved pain, function and anxiety, but not quality of life. Subgroup analysis reveals that XR‐based rehabilitation improved postoperative pain and function more significant within 1 month postoperatively.
Youssef et al.	Is virtual reality effective in orthopaedics rehabilitation? A systematic review and meta‐analysis	19 studies	‐	Clinical practice	Virtual reality	Rehabilitation	2019	Systematic review and meta‐analysis	For fibromyalgia and back pain, as well as after knee arthroplasty, the evidence of VR effectiveness compared with exercise is absent or inconclusive.
Wang et al.	Technology‐assisted rehabilitation following total knee or hip replacement for people with osteoarthritis: A systematic review and meta‐analysis	5 trials 232 participants (*n *= 232)	VR technology employed included Wii balance board, Wii game consoles, 3D avatar for robot‐assisted walking and virtual boat rowing	Clinical practice	Virtual reality	Rehabilitation	2019	Systematic review and meta‐analysis	VR was classified under ‘technology assisted rehabilitation’ In terms of mobility, technology‐assisted rehabilitation is not significantly superior to usual care post TKR Moderate‐quality of evidence showed technology‐assisted rehabilitation, particularly telerehabilitation, had a statistically significant improvement in pain; and low‐quality of evidence for the improvement in functional mobility in people undergoing TKR.
Byra et al.	The effectiveness of virtual reality rehabilitation in patients with knee and hip osteoarthritis	7 studies	VR technology employed included Nintendo Wii, biofeedback training, rehabilitation visualisation system	Clinical practice	Virtual reality	Rehabilitation	2020	Systematic review and meta‐analysis	There is no conclusive evidence that interventions based on VR are more effective than standard physiotherapy treatment in the rehabilitation of patients suffering from osteoarthritis, including patients after total knee arthroplasty. Interventions based on VR are promising in view of pain management, range of motion and proprioception.
Peng et al.	VR‐based rehabilitation in patients following total knee arthroplasty: A systematic review and meta‐analysis of randomised controlled trials	8 studies and 805 patients (systematic review) 7 studies (meta‐analysis)	VR‐based rehabilitation—Nintendo Wii Fit board, interactive VR telerehabilitation kit, VR equipment for rowing, use of avatar and real‐time feedback, VR glasses, VERA (Virtual Exercise Rehabilitation Assistant)	Clinical practice	Virtual reality	Rehabilitation	2022	Systematic review and meta‐analysis	VR‐based rehabilitation improved pain (VAS) and function (WOMAC and HSS) but not postural control (TUG) following TKA compared to conventional rehabilitation
Gazendam et al.	Virtual reality rehabilitation following total knee arthroplasty: A systematic review and meta‐analysis of randomised controlled trials	9 studies 835 patients	VR rehabilitation—Hardware/software devices creating a simulated environment for TKA patients	Clinical practice	Virtual reality	Rehabilitation	2022	Systematic review and meta‐analysis	No difference in pain scores between VR based and traditional rehabilitation at 2 weeks and 3 months post operatively VR based rehabilitation demonstrated improved function outcomes at 12 weeks and 6 months post operatively compared to traditional rehabilitation. One trial demonstrated significant cost savings with VR based rehabilitation
Iacono et al.	The use of augmented reality for limb and component alignment in total knee arthroplasty: Systematic review of the literature and clinical pilot study	Two preclinical studies Total knee arthroplasty patients (*n *= 5)	Knee+ augmented reality navigation	Clinical practice	Augmented reality	Surgery	2021	Systematic review and pilot study	AR Knee + system could perform a cutting error of less than 1° of difference about coronal alignment of femur and tibia and less than 2° about flexion/extension of femur and posterior tibial slope
Daniel et al.	Augmented reality for assistance of total knee replacement	Knee models	Using AR to display cut produced by comparing inclination angles of joint with cutting tools	Clinical practice education	Augmented reality Virtual reality	Surgery surgeons	2016	Mechanistic study	The system developed shows that it is possible to design systems based on machine vision and VR, to create platforms for training and support in surgical procedures, which in addition present a correct performance; its cost of production was lower compared to the one exposed in
Edwards et al.	Immersive VR enables technical skill acquisition for scrub nurses in complex revision total knee arthroplasty	Ten participants	Using immersive VR software—Nurses wearing VR headset and performing procedures using motion‐tracked controllers	Education	Virtual reality	Nurses	2021	Cohort study	Immersive VR training improves scrub nurses understanding, technical skills and efficiency in complex revision knee arthroplasty surgery. These iVR‐learnt skills appear to translate into the physical environment
Vestermark et al.	Cognitive training for robotic arm‐assisted unicompartmental knee arthroplasty through a surgical simulation mobile application	12 participants	Touch Surgery—mobile‐based app that combines cognitive task analysis with VR medium to familiarise user with surgical procedure through interactive rehearsal for the Mako robotic arm‐assisted UKA	Education	Virtual reality	Surgeons	2019	Cohort study	Touch surgery app was better than traditional paper‐based learning for both immediate posttest performance and long‐term information recall of Mako robotic arm‐assisted unicompartmental knee arthroplasty
Newman et al.	Content and face validity assessment of the sim‐K haptic‐feedback enhanced total knee replacement virtual reality simulator	30 participants	Ossim Sim‐K—first VR TKR simulator with haptic feedback	Education	Virtual reality	Surgeons	2018	Cohort study	Ossim Sim‐K attained positive responses for items related to both face and content validity
Hall et al.	Technology‐enhanced learning in orthopaedics: Virtual reality and multi‐modality educational workshops may be effective in the training of surgeons and operating department staff	13 surgical trainees (bootcamp) 15 participants (VR TKA simulation)—4 consultants, 6 surgical trainees, 5 scrub nurses	VR (Oculus Rift system) with ATTUNE Total Knee System (Johnson & Johnson Medical Devices) and saw‐bone simulation, tutorials and case‐based symposia	Education	Virtual reality	Surgeons, nurses	2023	Cohort study	VR‐mediated simulation could augment the education of surgical trainees and scrub team staff by improving comprehension of the surgical process map. Integrated multi‐modality ‘Bootcamp‐style’ training activities constructed around trainees’ needs may provide a sustainable solution to bridge the experience gap related to reduced exposure to elective orthopaedic practice.
McKinney et al.	Virtual reality training in unicompartmental knee arthroplasty: A randomised, blinded trial	22 participants	Medial unicompartment knee arthroplasty performed using Osso VR training goggles and headset versus industry standardised technique guide for Zimmer Persona medial partial knee arthroplasty system (Zimmer PPK)	Education	Virtual reality	Surgeons	2022	Randomised controlled trial	Residents who trained with the immersive VR executed more steps correctly and completed their training and the procedure in faster time and scored higher in all global assessment categories. These results are promising and demonstrate a clear role for immersive VR as a surgical training tool
Alpaugh et al.	Immersive technologies for total knee arthroplasty surgical education	‐	Applications of AR and VR in modern total knee replacement surgical education	Education	Augmented reality mixed reality virtual reality	Surgeons	2021	Review article	VR and AR are not only appealing from patient safety and resource utilisation perspectives but also because they have been shown to be efficacious in teaching basic procedural and technical knowledge. There is limited evidence on immersive technology for total knee replacement education.
Mandal et al.	Surgery training and simulation using virtual and augmented reality for knee arthroplasty	‐	Surgical care and surgical education relying on AR and VR	Education	Augmented reality mixed reality virtual reality	Surgeons	2022	Review article	Despite the increasing use of AR and VR in training and clinical services, further research is required to define how new technology would improve patient outcomes and determine whether all patients would benefit from it
Shaikh et al.	Exposure to XR and artificial intelligence‐based manifestations: A primer on the future of hip and knee arthroplasty	‐	Narrative review of XR orthopaedic ecosystem with emphasis on hip and knee arthroplasty	Research	Augmented reality mixed reality virtual reality extended reality	Artificial intelligence	2023	Narrative review	In a field where exposure is critical to clinical success, XR represents a novel stand‐alone software‐infused service that optimises technical education, execution, and expertise but necessitates integration with AI and previously validated software solutions to offer opportunities that improve surgical precision with or without the use of robotics and computed tomography‐based imaging.
Bagaria et al.	Robotic‐assisted knee arthroplasty (RAKA): The technique, the technology and the transition	‐	Analysis of the robotic‐assisted knee arthroplasty system and ideas for future integration with augmented and VR	Research	Augmented reality virtual reality	Robotic assisted	2020	Review article	The commitment of almost all major implant manufacturers in investing in robotics likely means that the evolution of robotic technology and this decade will be exciting with rapid strides revealing paradigm shift and evolution of technology with significant reductions of cost enabling it to be available universally.

*Note*: Colour code for the interpretation of the article's conclusion (Green = Favourable, Yellow = Ambivalent, Red = Unfavourable). Abbreviations: AI, artificial intelligence; AR, augmented reality; IVR, immersive virtual reality; MIS‐TKR, minimally invasive total knee arthroplasty; PT, physiotherapy; TKA, total knee arthroplasty; TKR, total knee replacement; UKA, unicompartmental knee arthroplasty; VR, virtual reality; VRT, virtual reality tools; XR, extended reality.

**Figure 3:**
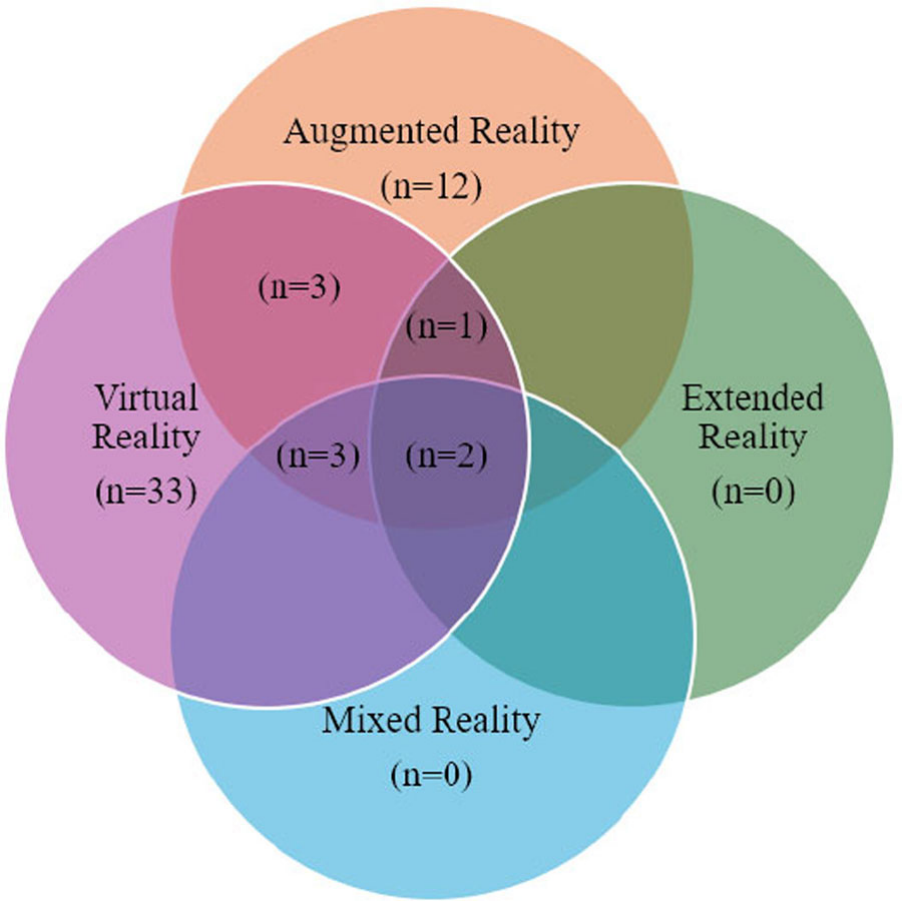
Summary of types of technology mentioned by the 54 articles.

Fifty‐four articles examined using XR, VR, AR, and MR in knee arthroplasty [1, 5, 8, 9, 11, 14, 16, 19, 23, 25, 26, 27, 33, 38, 40, 41, 42, 46, 47, 49, 50, 54, 55, 56, 57, 63, 64, 67, 71, 76, 84, 86, 89, 98, 100, 102, 104, 105, 108, 110, 111, 113, 114, 119, 122, 125, 129, 130, 131, 133, 136, 137, 141, 142]. The year of publication spanned from 2006 to 2024. Most of the articles mentioned the use of VR (*n *= 42). The remaining articles mentioned AR (*n *= 21), MR (*n *= 5) and XR (*n *= 3), with some articles writing about more than 1 type of technology within the same article (*n *= 9) (Figure 3).

**Figure 4:**
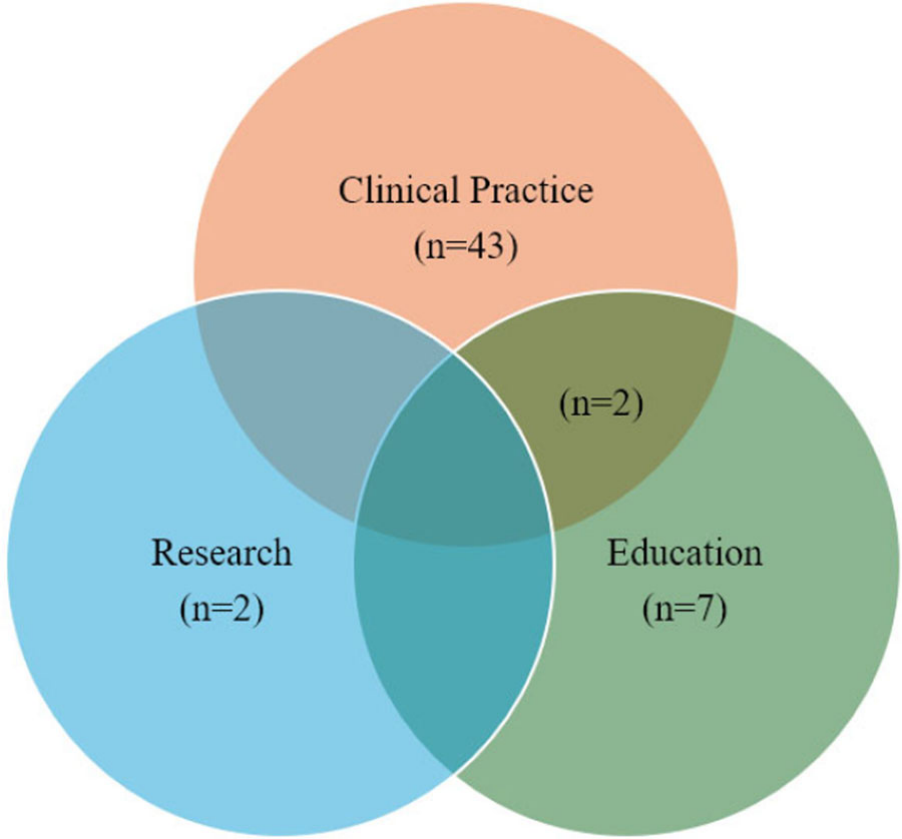
Summary of the subthemes of the 54 articles.

The articles were categorised into themes of ‘clinical practice’ (*n *= 45), ‘education’ (*n *= 9) and ‘research’ (*n *= 2), with two articles classified under more than one theme. The articles under the theme of clinical practice were further subdivided into themes of ‘anaesthesia’ (*n *= 5), ‘rehabilitation’ (*n *= 26) and ‘surgery’ (*n *= 14). The articles under the theme of ‘education’ were further subdivided into themes of ‘surgeons’ (*n *= 8) and ‘nurses’ (*n *= 2). The articles under the theme of ‘research’ could be classified into ‘robot‐assisted’ (*n *= 1) and ‘artificial intelligence’ (*n *= 1) (Figure 4).

There was a mixture of article types, ranging from cohort studies (*n* = 15), experimental studies (*n *= 2), mechanistic studies (*n *= 4), narrative review (*n *= 1), RCT (*n *= 16), retrospective study (*n *= 1), review articles (*n *= 3) and systematic reviews (and meta‐analysis) (*n *= 12).

### Clinical practice

#### Rehabilitation

Twenty‐six articles analysed the use of XR (*n *= 1), VR (*n *= 24) and AR (*n *= 4) in the rehabilitation of post knee arthroplasty patients [5, 16, 19, 23, 26, 27, 41, 42, 46, 47, 49, 54, 55, 56, 67, 76, 105, 110, 111, 114, 122, 125, 126, 137, 141, 142]. The article types were cohort studies (*n* = 4), RCT (*n *= 13) and systematic reviews (and meta‐analysis) (*n *= 9).

Thirteen articles demonstrated the effectiveness of VR rehabilitation postknee arthroplasty. These were in the form of outcome measures such as improvements in clinician‐patient interaction beyond the hospital setting, cost savings, convenience, at‐home monitoring, patient satisfaction, alleviating postoperative pain, reduced cost of medical transport, improved range of motion, accelerated functional recovery and improved mental health of patients [27, 76].

However, six articles reported that VR rehabilitation did not confer an improvement in the patient's quality of life [54, 125], functional weight‐bearing symmetry [26]. VR rehabilitation was also not superior in pain relief [47] and in improving functional outcomes compared to conventional therapy [23, 49, 137]. Furthermore, when compared to conventional therapy, seven articles reported no significant improvements in outcomes such as postural stability [55], pressure and spatiotemporal gait parameters [56], pain, anxiety and long‐term function [41] or balance [110], implying that VR rehabilitation could be used as an adjunct to conventional therapy without incurring the risk of poorer outcomes [19, 42, 142].

#### Surgery

Fourteen articles analysed the use of XR (*n *= 1), VR (*n *= 4), AR (*n *= 13) and MR (*n *= 2) in knee arthroplasty [11, 14, 25, 33, 40, 50, 64, 84, 108, 113, 129, 130, 131, 136]. The article types were cohort studies (*n *= 5), experimental studies (*n *= 2), mechanistic studies (*n *= 4) and systematic reviews (and meta‐analysis) (*n *= 3).

Eleven articles concluded that the use of AR‐guided navigation aided in the visualisation of the anatomy of the knee [50, 84], allowing for precise alignment and accurate coronal, sagittal and rotational cuts in TKA [14, 25, 50, 108, 130]. AR‐guided navigation resulted in more accurate distal femoral resections than with conventional intramedullary guides [131], demonstrated potential for future minimally invasive TKR [136] and allowed for a better measure of the effect of tibial rotation [40]. In terms of accuracy, knee arthroplasty with AR‐guided navigation displayed a cutting error of less than 1° of difference in the coronal alignment of femur and tibia and less than 2° in flexion/extension of the femur and posterior tibial slope [64]. Together with AI‐based tools, AR, VR and MR have enhanced decision making, surgical planning, and the precision and reproducibility of surgical procedures [11]. Furthermore, designing cost‐effective systems based on AI and VR for training and support in surgical procedures has been shown to be feasible [33, 50].

However, one article concluded that the clinical effectiveness of AR‐based navigation was not conclusive and lacked generalisability in unicompartmental knee arthroplasty [131]. One article reported some sagittal outliers in TKA alignment while using AR‐guided navigation [14]. One article had also reported that the use of VR to identify anatomical landmarks was also found to be unreliable [113].

#### Anaesthesia

Five articles analysed the use of VR in knee arthroplasty: [9, 63, 71, 100, 104] These articles were cohort studies (*n *= 2), RCTs (*n *= 2) and retrospective studies (*n *= 1).

The articles concluded that iVR when paired with spinal anaesthesia during TKA was associated with lower doses of intraoperative sedative usage [9], decreased intraoperative adverse events and increased postoperative comfort compared to spinal anaesthesia usage alone [104]. Furthermore, VR‐mediated analgesia might induce long‐lasting analgesia through more potentiated brain plasticity with improved active knee range of motion [72]. In preoperative adductor canal catheter insertion, VR distraction was concluded to decrease the use of intravenous opioids and sedatives. This has led to a decrease in procedure‐related pain by VR serving as a nonpharmacological alternative to intravenous sedation [100].

However, one article concluded that the use of iVR with spinal anaesthesia led to no difference in patient anxiety or patient satisfaction post TKA [104]. One article had concluded that there was no significant difference in patient‐controlled intra‐operative propofol during TKA with the use of iVR during regional anaesthesia [63].

### Education

#### Surgeons

Eight articles analysed the use of XR (*n *= 1), VR (*n *= 8), AR (*n *= 4) and MR (*n *= 3) in knee arthroplasty related to education for surgeons [1, 33, 50, 57, 86, 89, 98, 133]. The article types were cohort studies (*n *= 3), RCT (*n *= 1), review articles (*n *= 2), mechanistic studies (*n *= 1), systematic reviews (and meta‐analysis) (*n *= 1).

The articles concluded that AR and iVR had demonstrated the potential to improve the cost‐effectiveness of modern surgical training [33, 50]. Compared to traditional paper‐based learning, VR demonstrated better outcomes for immediate posttest performance and long‐term information recall in unicompartmental knee arthroplasty [133]. VR TKR simulator with haptic feedback has demonstrated favourable outcomes in both face and content validity assessments [98]. In unicompartmental knee arthroplasty, residents who practised using iVR demonstrated that they completed more steps correctly and finished their procedure faster, scoring higher in all global assessment categories [89]. VR‐mediated simulations with ancillary staff could also improve the knowledge of surgical trainees by enhancing their understanding of the surgical process map [57].

However, it has been noted that there might be limited evidence on the use of AR and iVR in TKR education [1] and that further research is required to determine if this technology could improve patient outcomes [86].

#### Nurses

The two articles under this category were both cohort studies. Both articles concluded that iVR improves the comprehension, surgical skills and efficiency of scrub nurses in complex revision knee arthroplasty and TKA [38, 57]. iVR has been shown to improve nursing staff confidence and minimise anxiety, while acquiring the required technical skills to perform in the operating theatre efficiently [38].

#### Research

Two articles analysed the use of XR (*n *= 1), VR (*n *= 2), AR (*n *= 2), and MR (*n *= 1) in the research and future direction of knee arthroplasty [8, 119]. The article types were narrative review (*n *= 1) and review article (*n *= 1).

Both articles envisioned the future of XR, VR, AR and MR in knee arthroplasty involving integration with AI [119] and robotic‐assisted surgery [8]. It is argued that XR requires a union with AI and other previously validated software to enhance technical education and improve surgical accuracy [119]. Furthermore, given the high investment and interest level of major implant manufacturers in the field of robotics, robotic surgery is postulated to flourish with significant surgical cost savings. Related technologies such as XR are likewise anticipated to thrive, fostering greater adoption in the future [8].

## DISCUSSION AND FUNDING

This scoping review focused on mapping out the literature on the emerging topic of using XR, VR, AR and MR in knee arthroplasty and identifying gaps to guide future research in this field. In this scoping review, we identified 54 articles addressing the use of XR, AR, VR and MR in clinical practice, education and research of knee arthroplasty from 2006 to 2024.

### Clinical practice

#### Rehabilitation

The conclusions from the articles (*n *= 26) under the subtheme of rehabilitation displayed the most ambivalence compared to all other subthemes. The articles were published between 2012 and 2024. Half of the articles under this subtheme either concluded that XR, VR, AR or MR technology did not improve rehabilitation outcomes or had no difference compared to conventional therapy.

The latest article published (at the point of writing this scoping review) in the field of rehabilitation involving the use of XR, AR, VR and MR in the field of arthroplasty belonged to Su et al. [125]. The article was a meta‐analysis and systematic review of 14 RCTs and 989 patients [125]. Before the publication of this article, some articles had acknowledged the potential use of such technology in knee arthroplasty. However, stronger scientific evidence is required to substantiate its real‐world application [55].

From the article, the measured outcomes of the meta‐analysis and systematic review were categorised under the headings of pain scores, functional outcomes, range of motion, timed up‐and‐go test and anxiety score. The data concluded that XR‐based rehabilitation improved pain, function and anxiety, but not quality of life. However, due to the small number of included studies and sample size, more high‐quality RCTs are required to validate the impact of XR‐based rehabilitation on anxiety and quality of life [125].

Beyond the efficacy of XR technology in rehabilitation, the success of postoperative rehabilitation hinges on other factors such as effective pain management, patient motivation and adherence and skilled physiotherapy support [41]. Unfortunately, physiotherapists are scarce, particularly in developing countries and rural areas [24]. Even if a physiotherapist is present, not all patients might be able to afford the high cost of physiotherapy [111]. Additionally, with the recent coronavirus disease 2019 (COVID‐19) pandemic, face‐to‐face physiotherapy has been more inconvenient in inpatient and outpatient settings [70]. Therefore, further efforts should also focus on overcoming these challenges, in addition to the XR technology itself, to improve patient rehabilitation outcomes.

An area of research that future XR‐related technology should focus on is the use of MR in knee arthroplasty‐related rehabilitation. There are many articles regarding AR and VR for rehabilitation postknee arthroplasty, but no articles have been found regarding the use of MR for rehabilitation. The advantage of using MR is the user's real‐time interactivity with the environment generated from VR and AR, enhancing the user's engagement with the activity [126]. Alongside XR technology, advances in other computer‐assisted technology, such as biofeedback implementations into hardware platforms and biofeedback augmented motion capture, could also be investigated in the future [23].

For a rehabilitation system to be strongly supported, it must be practical and accessible, provide high patient and clinician satisfaction, demonstrate clinical effectiveness and remain cost‐effective [68]. Efforts should be made to improve patient satisfaction by overcoming some of the current side effects from the use of VR devices for rehabilitation, such as cybersickness. Cybersickness can cause nausea, vertigo, headache, giddiness and increase the risk of accidental collision with nearby objects [51, 139].

Apart from research into the cost‐effectiveness and efficacy of XR technology, further research should also be directed to overcoming challenges that might prevent XR technology from improving patient outcomes. One such challenge is the poor engagement rate of the elderly. The elderly form a significant proportion of knee arthroplasty patients who use XR technology for postoperative rehabilitation [126]. Future efforts should be directed to improving the engagement of the elderly to improve their patient rehabilitation outcomes.

#### Surgery

The articles (*n *= 14) under this subtheme were from 2006 to 2024. Unfortunately, of the 14 articles, most were mechanistic, cohort, or experimental studies. No RCTs were included under the subtheme of surgery in this scoping review. Clinical studies are required to assess the effectiveness of novel XR‐guided navigation systems in knee arthroplasty and its impact on cost‐effectiveness and patient outcomes [11, 50].

There was a wide range of sample sizes (*n *= 5 to *n *= 76) from the articles included in the scoping review. The results from the small sample sizes in some of the articles might need to be more reliable and accurate when applied to the general population. Furthermore, the accuracy of the proposed AR system used in the studies could have been heavily influenced by the surgeon's experience and skills involved in the study, possibly affecting the replicability of its findings [50].

Despite multiple articles illustrating the effectiveness of AR and VR guided navigation systems, future research in this subtheme should focus on resolving the persistent limitations in this technology. These limitations include poor image registration, angular deviation, inaccurate depth perception, long processing time, risk of occlusion, cybersickness, poor tactile feedback, device‐related issues and the high cost of technology [84]. Of these challenges, enhancing tactile feedback remains the most formidable challenge, with progress largely dependent on advances in computer power and software optimisation [50].

Before the advent of AR‐guided navigation in knee arthroplasty, intraoperative cuts adhered strictly to pre‐operative measurements via technology such as robot‐assisted surgery [115] or computer‐assisted navigation technology [28]. However, the high operating costs of this technology limited its use to only a few surgical centres [39]. AR navigation‐guided knee arthroplasty might be feasible for ambulatory surgery centres. AR navigation equipment is more compact and straightforward than traditional computer‐assisted systems, hence reducing the need for substantial capital investment and high maintenance cost. Furthermore, AR navigation equipment is smaller and more portable, thus lowering the equipment's footprint in the operating theatre [15].

#### Anaesthesia

The articles (*n *= 5) included under the subtheme of anaesthesia are more recent, dating from 2017 to 2022, which consists of two RCTs.

All five articles in the scoping review under this subtheme used VR technology. The limitations with VR technology regarding anaesthesia include the battery usage (with some headsets turning off due to low battery) and the technical headset (the headset needed to be set up correctly in some patients) [104].

Future research in this subtheme could focus on incorporating AR and MR into the field of anaesthesia. Some patients (*n *= 9/25) were not compliant with the virtual headset throughout the operation, possibly because they did not feel completely engaged with the VR setting [63]. As an example of improving patient engagement, the University of Washington had developed a Snow World VR software that promotes further engagement with the user by allowing the user to throw snowballs in the virtual environment [60]. Further research should be directed to improve such software by including haptic feedback and integration with AR by incorporating an overlay onto the existing real environment. This could improve analgesic outcomes by enhancing patient engagement and compliance.

### Education

#### Surgeons

Traditionally, dissection models have been considered the gold standard in surgical teaching due to the supposed benefit of increased authenticity and precision with respect to real tissue feel. However, there needs to be more conclusive evidence that this would translate to possessing a technical aptitude in real‐life operating contexts [50, 86]. XR technology, iVR in particular, provides a platform for surgeons to improve their surgical understanding and skills by simulating various surgical scenarios as training [50]. Despite this, although not reflected by the data gathered in our scoping review for knee arthroplasty, an article focused on assessing the efficacy of VR training for total hip arthroplasty revealed that two participants could not achieve competence despite undergoing VR training [79]. This could imply that VR education might not be suitable for all surgical trainees and its effectiveness may depend on factors such as digital literacy, adaptability to new technology and differences in attitude towards XR amongst young and more senior surgeons [119].

The effectiveness of using XR technology as a training platform for surgeons was highlighted during the recent COVID‐19 pandemic, during which there was up to 50% reduction in surgical trainees’ logbook numbers [30]. During this time, VR training had formed a crucial part of the training curricula. Future applications of XR technology for education could include more efficient promulgation of data by transcending operational and geographic constraints, thereby addressing the global access gap to high‐quality surgical training [86]. For example, surgeons in developing countries could utilise training platforms to enhance their surgical skills without the cost of travel or expensive dedicated training facilities. Furthermore, immersive conferences can be conducted internationally, allowing for discourse with field experts without the need for physical travel [1].

**Figure 5:**
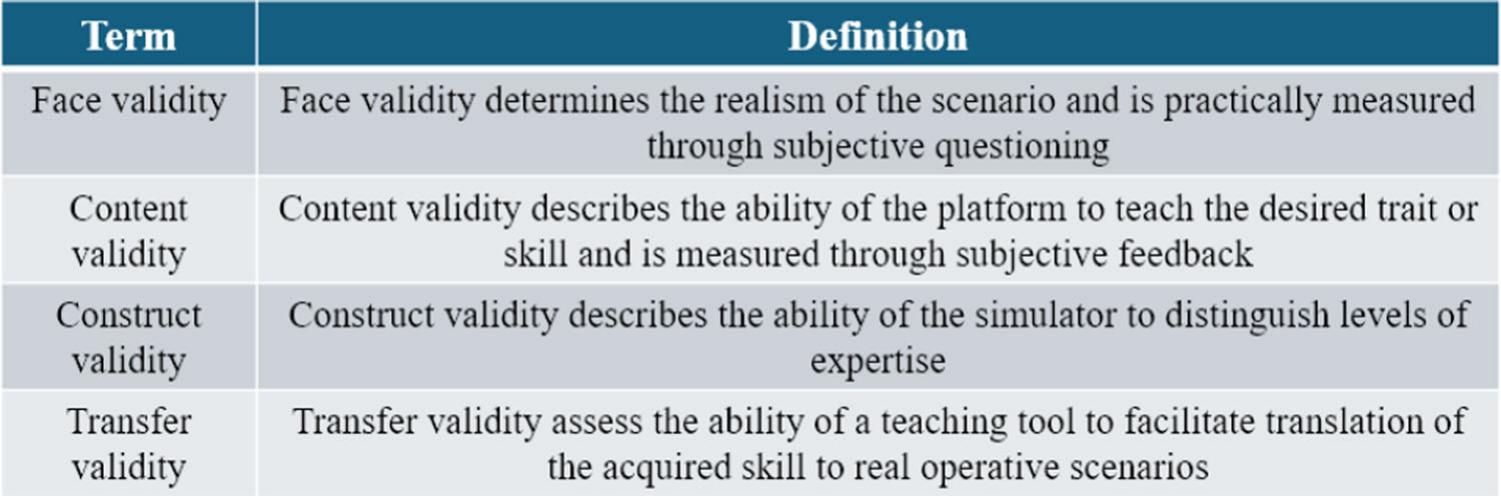
Definition of various forms of validity [50].

Of the articles reviewed under this subtheme, only three mentioned the terms ‘face validity’ and ‘content validity’. These terms are used in the scientific evaluation of the performance of an XR technology platform for education (Figure 5). Face validity refers to the authenticity of the experience, while content validity is the assessment of the educational capability of the platform [98]. Given that the purpose of educational simulators is to transfer operating skills acquired from the simulator onto the surgeon, the most crucial of the concepts would be transfer validity [81]. There are numerous objective assessments and scales to measure transfer validity, such as the Objective Structured Assessment of Technical Skills (OSATS), the global ratings scale of performance, and the arthroscopic surgical skill evaluation tool. Using these terms and objective measures should become more commonplace in future research regarding the effectiveness of XR technology for educational purposes.

The use of XR technology could allow trainees to improve their skills in procedures that are less common and more complex. The estimated failure rate after TKA is about 1% annually, with prosthesis loosening accounting for 22.8 to 31.2% of cases [77]. Opportunities to practice technical and intra‐operative surgical skills for revision arthroplasty are limited to curated fellowship training or individualised practice [50]. Furthermore, revision arthroplasty is associated with poorer functional outcomes and higher risks of complications [50]. Apart from revision arthroplasty, robotic arm‐assisted unicompartmental knee arthroplasty is a surgery that might be intimidating for a surgical resident or trainee to comprehend and perform [133]. Therefore, future research in the education subtheme should exploit XR technology's capabilities and provide opportunities for surgeons to enhance their skills in revision arthroplasty and complex procedures like robotic arm‐assisted unicompartmental knee arthroplasty.

#### Nurses

In the operating theatre and ambulatory surgical centres, it has been concluded that poor teamwork behaviour by the operating theatre teams was associated with an increased chance of the patient experiencing death or significant complications [87]. Specific to arthroplasty, an operating team efficiency is essential in improving patient outcomes as extended operating times are associated with a higher risk of operative complications [20, 112, 123]. To improve procedural efficiency, surgical staff must possess intricate technical knowledge of surgical inventory, especially for complex procedures such as revision TKA with inventories exceeding 500 pieces of equipment [4]. Hence, iVR is a cost‐effective solution that ensures scrub nurses are adequately prepared for these complex operations [57, 79].

Apart from scrub nurses, other ancillary operating staff members can benefit from using XR technology. Surgical device representatives have a considerable role in improving the efficiency of the operating theatre [82]. Regulating the training of ancillary surgical staff as a holistic unit might result in developments in knowledge and efficiency in the operating theatre [80]. This could be achieved by synchronising multiple VR sets to achieve a combined VR experience that could enhance overall training experience, augment multidisciplinary team‐based practice and streamline surgical workflows [34]. Future research in this subtheme should consider the effects of XR technology in the education of ancillary staff extending beyond scrub nurses and surgical devices representatives to include surgical radiographers, anaesthetists and even medical students.

In this scoping review, there were two articles regarding the use of VR in education for scrub nurses, of which both articles concluded that VR is an excellent tool to improve the knowledge and comprehension of scrub nurses. However, there is a lack of data in terms of the objective evaluation of the impact of XR technology‐related training on objective indicators of performance in the operating theatre. Examples of such objective indicators include time taken for each procedural step, economy of movement (such as hand movements or path length) and objective measures such as OSATS [57]. Future research in this subtheme should therefore focus on these objective evaluations.

### Research

#### Artificial intelligence

Two nascent and transformative technologies have recently garnered significant interest in orthopaedics—XR and AI [119]. XR's potential would be significantly hampered without the use of AI. Computer vision (CV), a subset of AI, serves as ‘eyes’ for the computer, capturing and refining visual data for the computer to translate [94], thereby complementing the XR software by allowing it to interpret, evaluate and manipulate the data. The data can then be broadcast and relayed to the surgical team via XR technology. This permits real‐time feedback and allows for the verification of data against digital surgical guides and imaging to prepare for the subsequent steps of the operation [97].

The proposed synergy between AI and XR can be appreciated in future knee arthroplasty. An AI‐based software is used to register and calibrate anatomic surface landmarks on the exposed knee via surface mapping based on a library of ‘normal’ computed tomography (CT) images of the human knee. After registration, data such as resection depths and axis can be obtained with reasonable accuracy, thus removing the need for preoperative CT scans and its associated increased cost and radiation exposure. Combining CV and XR, a surgeon's mechanical predilections and workflow can be assessed to systematise intraoperative focus and practice digitally. This would allow for an ordered collection of the surgeon's personalised operative steps for display to ancillary staff to enhance team efficiency. Digital mentorship could occur for complex cases with the mentor's ordered collection of workflows easily accessible intraoperatively [119].

An example of the synergy between XR and AI in knee arthroplasty can be appreciated in Medacta's NextAR (Medacta) with its capability of assessing 3D soft‐tissue activity instantaneously [92]. Data from the patient's preoperative CT scan is transferred virtually to smart glasses and overlaid onto the patient. A tracker is anchored in the necessary measurement area. The tracker can convey its spatial position in 6 degrees of freedom and limit error to less than 0.5 degrees. Data regarding tension in the cruciate and collateral ligaments is readily available upon placing the implant trial on the patient [119].

Although the most desirable implant design and alignment remains unclear, XR and AI can unify soft‐tissue anatomy with biomechanical features through an XR implant design system. This system allows pre‐operative simulation of physiological conditions and intraoperative simulation of the ideal postoperative implant position. This was observed in trauma via preoperative designing of osteosynthesis plates in unilateral pelvic and acetabular fractures [121]. The system processed data from preoperative CT scans and virtually reduced the fractures. Based on the virtual fracture reduction, an ideal curve was designed to analyse and produce the required implant model, while adjusting the implant based on the dimensions from the analysis. This allowed surgeons to manipulate the osteosynthesis plates preoperatively according to the fracture pattern, thus shortening operating time and relying on virtual planning to guide the surgical approach [119].

#### Robotic assisted surgery

More than 15% of unicompartmental knee arthroplasty in the USA was performed in 2023 using a robotic platform [58]. This number is expected to grow soon due to increased surgeon knowledge and competence, increased training opportunities in robotic surgery, higher patient demand and increased affordability of robotic surgery platforms. Surrogate markers of growth in robotic surgery have been promising. These markers include an increase in patents filed and peer‐reviewed publications [21, 32]. The goals of robotic surgery are primarily aligned with that of knee arthroplasty—improved operating time, depreciation of instrumentation and equipment and reproducing repeatable outcomes regarding precise alignment and enhanced soft tissue balancing [32, 65, 83].

Alongside the use of haptic boundary and live feedback, robotic surgery has also been associated with MR [8]. This use of MR allows the surgeon to interact with both the virtual and real‐time interface, empowering the surgeon to control the robotic instruments [8]. Future research integrating XR and robotics‐assisted surgery should delve into alternative patient requirements, outcomes and the following generation implant design perspectives [8]. Given that most large implant companies have invested heavily in robotic technology prospects in the future [114], we would expect future research to focus on using XR technology to complement this expected development in robotic technology.

### Limitations

This scoping review has some limitations. Since 1992, the nomenclature of AR, VR and, more recently, MR and XR‐related technologies has varied and evolved [69]. Some studies in the past might have used XR technology without explicitly using the specific terms AR, VR, MR or XR. These articles might not have been captured by our literature review.

The scoping review was a significant undertaking, and its results are only up to date as of April 2024. Only articles written in English were included in this scoping review.

## CONCLUSION

This scoping review focused on mapping the literature on the emerging topic of using XR, VR, AR and MR in knee arthroplasty in terms of how it is used and to identify gaps to guide future research in this field.

Articles under the subtheme of ‘rehabilitation’ demonstrated the most ambivalence in terms of the effect of XR, VR, AR and MR technology post knee arthroplasty, while most articles under the remaining subthemes of ‘surgery’, ‘anaesthesia’, education for ‘surgeons’, ‘nurses’ and future ‘research’ generally demonstrated practical usage of XR, VR, AR and MR technology and paints a promising future for this technology in knee arthroplasty.

Future research efforts should be focused on the following:

1. Exploring the usage of MR in rehabilitation for knee arthroplasty.
2. Overcoming the side effects and challenges of the use of XR‐related technology.
3. Conducting RCTs regarding the use of XR‐related technology in the subtheme of ‘surgery’ for knee arthroplasty.
4. Improving patient engagement and compliance with the use of XR‐related technology intraoperatively as anaesthesia.
5. The increased usage of standardised terms such as face validity, content validity, construct validity and transfer validity in assessing the use of XR‐related technology as an education tool.
6. Exploit capabilities of XR‐related technology in education for complex procedures such as revision arthroplasty or robotic arm‐assisted unicompartmental knee arthroplasty.
7. The use of objective indicators, such as OSATS, when assessing the impact of XR‐related technology on training staff.
8. Integration of XR‐related technology with AI.
9. Integration of XR‐related technology with Robotic Assisted Surgery.

There is much potential for using XR in knee arthroplasty in the future.

## AUTHOR CONTRIBUTIONS

**Ritesh Zun Xiong Deo**: Data curation; formal analysis; investigation; methodology; project administration; resources; writing. **Trishan Manav Sri Ram**: Investigation; resources. **Hamid Rahmatullah Bin Abd Razak**: Conceptualisation; supervision. **Nanne Kort**: Conceptualisation; supervision.

## CONFLICT OF INTEREST STATEMENT

The authors declare no conflicts of interest.

## ETHICS STATEMENT

The authors have nothing to report.

## Supporting information

Appendix A.

## Data Availability

Encourages data sharing.
