# Supplementary material for: Use of extended reality, virtual reality, augmented reality and mixed reality in clinical practice, education and research in knee arthroplasty: A scoping review
Source: J Exp Orthop. 2026 Jun 16;13(2):e70788. doi: 10.1002/jeo2.70788 (PMC13270410; doi:10.1002/jeo2.70788)
Supplement: Supplementary file 1 — Appendix A. [file JEO2-13-e70788-s001.docx]

**Appendix A**

**Cochrane Central Register for Controlled Trials**

(Search 1) (virtual reality knee arthroplasty):ti,ab,kw

(Search 2) (augmented reality knee arthroplasty):ti,ab,kw

(Search 3) (mixed reality knee arthroplasty):ti,ab,kw

**Google Scholar**

(Search 1) allintitle: virtual reality knee arthroplasty "virtual reality"

(Search 2) allintitle: virtual reality knee replacement "virtual reality"

(Search 3) allintitle: augmented reality knee arthroplasty "augmented reality"

(Search 4) allintitle: augmented reality knee replacement "augmented reality"

(Search 5) allintitle: mixed reality knee replacement "mixed reality"

(Search 6) allintitle: mixed reality knee arthroplasty "mixed reality"
